# Dimeric CCK2R radiotheranostic tracers synergize with mTOR inhibition for enhanced tumor therapy

**DOI:** 10.7150/thno.117021

**Published:** 2025-08-16

**Authors:** Linjie Bian, Zheyi Wang, Panli Li, Simin He, Jianping Zhang, Xiaoping Xu, Xiangwei Wang, Shaoli Song

**Affiliations:** 1 Department of Nuclear Medicine, Fudan University Shanghai Cancer Center; Department of Oncology, Shanghai Medical College, Fudan University, shanghai 200032, China.; 2 Shanghai Engineering Research Center of Molecular Imaging Probes, Shanghai 200032, China.; 3 Center for Biomedical Imaging, Fudan University; Shanghai 200032, China.; 4 Key Laboratory of Nuclear Physics and Ion-beam Application (MOE), Fudan University, Shanghai 200433, China.; 5 Department of Thoracic Surgery and State Key Laboratory of Genetic Engineering, Fudan University Shanghai Cancer Center, shanghai 200032, China; 6 Institute of Thoracic Oncology, Fudan University, Shanghai, China.

**Keywords:** Neuroendocrine tumors, Peptide receptor radionuclide therapy (PRRT), Multimeric radioligand, Oxidative stress modulation, Radiosensitization

## Abstract

**Purpose:** The cholecystokinin-2 receptor (CCK2R) is highly expressed in several neuroendocrine cancers, particularly in medullary thyroid carcinoma (MTC) and small cell lung cancer (SCLC) and represents a promising target for radiotheranostic applications. Several minigastrin-derived analogs, such as DOTA-MGS5 and DOTA-CCK-66, have demonstrated favorable tumor targeting and imaging performance. Building on these advances, we developed and evaluated a novel dimeric CCK_2_R-targeted radiotracer, and further investigated its radiosensitization potential in combination with mTOR inhibition.

**Experimental Design:** We designed a dimeric CCK_2_R-targeted agent, DOTA-CCK_2_R-dimer, labeled with ^68^Ga for PET imaging and ^177^Lu for radionuclide therapy. Furthermore, we combined [^177^Lu]Lu-DOTA-CCK_2_R-dimer with the mTOR inhibitor RAD001 and used single-cell RNA sequencing (scRNA-seq) to investigate the mechanisms of radiosensitization.

**Results:** Compared with its monomeric counterpart [^68^Ga]Ga-DOTA-CCK-66, [^68^Ga]Ga-DOTA-CCK_2_R-dimer demonstrated superior tumor targeting* in vivo*. Tumor uptake reached 26.13 ± 6.21 %ID/g at 2 h post-injection, which was significantly greater than that of the monomeric tracer (19.63 ± 3.35 %ID/g, *p* < 0.05). Additionally, [^177^Lu]Lu-DOTA-CCK_2_R-dimer selectively eliminated highly proliferative and poorly differentiated tumor cell subpopulations. Combination treatment with RAD001 improved therapeutic efficacy by suppressing glutathione-mediated detoxification and increasing oxidative stress. Furthermore, glutathione S-transferase kappa 1 (GSTK1) was identified as a key regulator that modulates radiosensitivity.

Conclusions: DOTA-CCK_2_R-dimer exhibits favorable *in vivo* stability, notable tumor retention, and excellent imaging performance. Combining this agent with mTOR inhibition offers a synergistic strategy to sensitize tumors to radiotherapy, providing a promising approach for treating refractory CCK_2_R-positive malignancies.

## Introduction

Cholecystokinin-2 receptor (CCK_2_R), a member of the G-protein coupled receptor (GPCR) family, is a membrane-bound transmembrane protein that is highly expressed in various neuroendocrine tumors (NETs), including those of small cell lung cancer (SCLC), and medullary thyroid carcinoma (MTC), while exhibiting low expression in most normal tissues 1-4. Owing to its high tumor specificity and accessibility on the cell surface, CCK_2_R has emerged as an ideal molecular target for peptide receptor radionuclide therapy (PRRT). In this approach, radiolabeled peptide ligands targeting CCK_2_R can selectively accumulate in tumor regions and serve both diagnostic and therapeutic purposes: ^68^Ga, a positron-emitting radionuclide, enables high-resolution positron emission tomography (PET) imaging for accurate tumor localization, whereas ^177^Lu, a β-particle emitter, induces DNA single-strand breaks, thereby enabling targeted radiotherapy and promoting tumor cell death 5, 6.

The first CCK_2_R-targeted radiotracers were primarily based on the endogenous ligand gastrin, particularly its essential C-terminal tetrapeptide binding motif (Trp-Met-Asp-Phe-NH_2_), which is recognized as the critical sequence required for receptor interaction 7. To improve pharmacological properties, synthetic derivatives of gastrin were designed, among which minigastrin, a truncated variant retaining the key binding sequence, has been widely employed as a scaffold for probe development. For example, PP-F11N and CP04, derivatives of minigastrin, incorporating a hexa-D-glutamate (D-Glu) sequence at the N-terminus to increase aqueous solubility and receptor binding affinity, were among the first CCK_2_R-targeted tracers to enter clinical evaluation 8. However, these tracers exhibit metabolic instability due to enzymatic cleavage of labile bonds, such as Tyr-Gly and Gly-Trp bonds, which compromises their *in vivo* durability and therapeutic efficacy. To address these limitations, N-methylated and nonnatural amino acids have been introduced into subsequent analogs such as DOTA-MGS5, significantly improving receptor affinity and reducing renal retention, making DOTA-MGS5 one of the most widely investigated CCK_2_R-targeted tracers 1. Building on these advancements, the scaffold was further optimized by replacing the N-terminal *H*-Glu-Ala-Tyr-Gly sequence with γ-D-Glu-PEG_3_ to yield DOTA-CCK-66, which exhibited increased metabolic stability and urinary clearance while maintaining excellent tumor targeting and imaging performance 4. Encouraged by these improvements in monomeric tracers, limited but promising efforts have also focused on dimerization strategies to further enhance receptor binding and tumor retention. For example, Sosabowski et al. designed a bivalent radiotracer based on the C-terminal motif of minigastrin, which exhibited improved tumor accumulation and retention *in vivo*. However, the renal uptake and nonspecific distribution of such dimeric constructs may also increase, underscoring the importance of balancing structural complexity with favorable pharmacokinetics 9.

In parallel, to further enhance the efficacy of PRRT, combination strategies incorporating pathway-specific inhibition have been investigated 10. Given the role of mTOR signaling in the regulation of tumor cell metabolism and radioresistance, mTOR inhibitors have been explored as potential radiosensitizers for combination therapy. Notably, Grzmil et al. reported that the mTOR inhibitor RAD001 (everolimus) potentiated the antitumor effects of [^177^Lu]Lu-PP-F11N, suggesting synergistic therapeutic potential 11. Nevertheless, although the efficacy of such combination treatment has been preliminarily demonstrated, the underlying molecular mechanisms remain insufficiently understood.

Therefore, we designed and synthesized a novel dimeric CCK_2_R-targeted radiotracer, the DOTA-CCK_2_R-dimer, based on the basis of the previously reported DOTA-CCK-66 scaffold. This probe was designed to increase the flexibility of the linker via a strategy that we refer to as a “conformation-unrestricted” design principle. In this approach, a flexible alkyl and polyethylene glycol (PEG) spacer is introduced between the chelator and the targeting peptide to preserve molecular conformational adaptability, reduce steric hindrance, and support native pharmacophore-receptor interactions. This approach differs from the conventional “conformation-restricted” approach, in which radionuclides are rigidly tethered to the pharmacophore, which constrains the spatial conformation of the entire probe. We systematically evaluated the physicochemical properties, biodistribution, and imaging performance of the resulting construct for direct comparison with [^68^Ga]Ga-DOTA-CCK-66 to assess structural and functional differences. Furthermore, we investigated the potential synergistic effects of RAD001 combined with [^177^Lu]Lu-DOTA-CCK_2_R-dimer *in vivo*. To elucidate the underlying mechanisms, we performed single-cell RNA sequencing (scRNA-seq) analysis on tumor samples derived from AR42J xenograft model mice, a well-established *in vivo* model of CCK_2_R-overexpressing tumors. This high-resolution transcriptomic approach enabled us to explore the cellular heterogeneity and identify the alterations in gene expression in response to mTOR inhibition, thereby providing mechanistic insights into the enhanced therapeutic response.

**Scheme 1:**
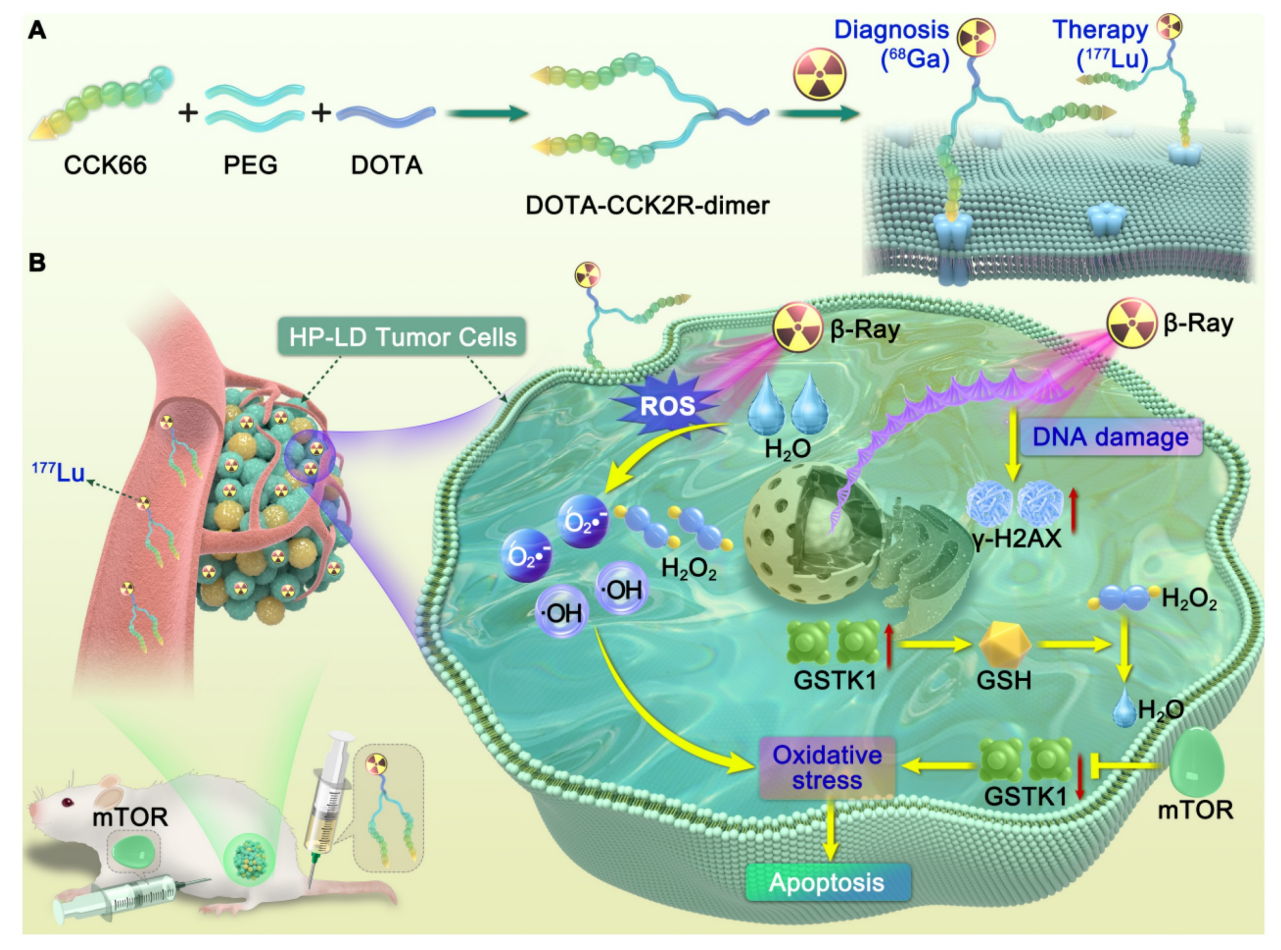
** Illustration of the design and mechanism of action of the dimeric CCK_2_R-targeted radiotracer combined with an mTOR inhibitor.** (A) Schematic of the structure of the [^68^Ga/^177^Lu]DOTA-CCK_2_R-dimer radiotracer. The DOTA-CCK_2_R dimer was designed on the basis of the DOTA-CCK-66 monomer structure. Two CCK-66 monomers were symmetrically linked via a γ-D-Glu residue and a flexible PEG_3_ spacer, yielding the dimeric structure. This design ensures appropriate spacing between the two pharmacophores, matching the distance between the binding sites of the CCK_2_R receptor dimer, thus enabling efficient multivalent binding and enhanced tumor-targeting capability. The N-terminus of the L-Glu residue is conjugated to the chelator DOTA, facilitating coordination with radionuclides and allowing radiolabeling with ^68^Ga for PET imaging or ^177^Lu for targeted radionuclide therapy (TRT). (B) Proposed mechanism of the selective elimination of high-proliferation, low-differentiation (HP-LD) tumor cell populations by [^177^Lu]Lu-DOTA-CCK_2_R-dimer. After binding to CCK_2_R-positive tumor cells, ^177^Lu emits β-rays that induce the production of intracellular reactive oxygen species (ROS), triggering oxidative stress and activation of the DNA damage response (γ-H2AX). The mTOR inhibitor downregulates GSTK1, disrupts the glutathione (GSH)-mediated detoxification pathway, and impairs cellular antioxidant defenses, thereby enhancing radiosensitivity and ultimately promoting tumor cell apoptosis.

Overall, this study establishes an integrated research framework from structural optimization to mechanistic exploration, offering new experimental evidence and theoretical guidance for the translational development of CCK_2_R-targeted radiotheranostics and combination therapy strategies **(Scheme 1)**.

## Materials and Methods

### Chemicals and Instruments

Detailed information on the chemical reagents, instruments, and cell lines used in this study is provided in **Supplementary Methods Section S1**.

### Molecular Docking Studies

The human (*Homo sapiens*) cholecystokinin B receptor crystal structure (PDB ID: 7F8V) with a resolution of 3.30 Å was downloaded from the RCSB PDB database and imported into AutoDockTools software (version 1.5.7, Scripps Research Institute, USA) for preparation, where unmutated chain E [auth R] was preprocessed on the basis of amino acid residues 0-465. The structures of CCK-66 and DOTA-CCK-66 were subsequently imported into Open Babel software for ligand preparation. Molecular docking to simulate the interactions between the compounds and the cholecystokinin B receptor was performed using AutoDock Vina. The docking results were analyzed with PyMOL (version 2.4.0, Schrodinger LLC, USA), with a focus on the three-dimensional binding modes between the cholecystokinin B receptor and CCK-66.

### Synthesis and Labeling Procedures

DOTA-CCK-66 (C_67_H_96_N_12_O_21_, MW = 1404.6813 Da) and DOTA-CCK_2_R-dimer (C_123_H_171_N_21_O_37_, MW = 2534.2145 Da) were synthesized by Chinese Peptide Co., Ltd. (Hangzhou, China) and used exclusively for research purposes.

For ^68^Ga labeling, 30 µg of precursor (prepared at a concentration of 1 µg/µL) was added to 110 µL of 1.5 M sodium acetate (NaOAc) solution, followed by the addition of 1 mL of [^68^Ga]GaCl_3_ eluate (370 MBq/mL). The reaction mixture was incubated at 75 °C for 20 min. The mass of the precursor was fixed at 30 µg was to standardize the radiolabeling conditions across all the experiments. For ^177^Lu labeling, 30 µg of DOTA-CCK_2_R-dimer (approximately 12 nmol) was mixed sequentially with 10 µL of [^177^Lu]LuCl_3_ solution (37 MBq/µL), 10 µL of 0.5 M sodium acetate (NaOAc), and 20 μL of 0.05 M hydrochloric acid (HCl), and the reaction mixture was incubated at 75 °C for 30 min.

### *In Vitro* Experiments

The hydrophilicity of [^68^Ga]Ga-DOTA-CCK_2_R-dimer and [^68^Ga]Ga-DOTA-CCK-66 was assessed by determining their partition coefficients (Log D_7.4_) in a phosphate-buffered saline (PBS; pH 7.4)/n-octanol (1:1, v/v) system. Briefly, ~37 kBq of each radiotracer was mixed with 2 mL of the biphasic system, vortexed for 5 min, and centrifuged at 15,000 × g for 5 min. The radioactivity in 100 μL aliquots of each phase was quantified using a γ-counter (n = 5).

*In vitro* stability was evaluated by incubating ~37 kBq of each radiotracer in PBS or human serum at 37 °C (n = 3). Samples were collected and analyzed by radio-high-performance liquid chromatography (radio-HPLC) at predetermined time points: 60, 120, and 180 min for ^68^Ga-labeled compounds and 1, 4, 24, 48, and 96 h for [^177^Lu]Lu-DOTA-CCK_2_R-dimer. To further assess the *in vivo* stability of [^177^Lu]Lu-DOTA-CCK_2_R-dimer, mice (n = 3) were intravenously injected with 3.7 MBq of the radiotracer in 200 μL of saline. Urine samples were collected at 1, 4, and 24 h post-injection, diluted, filtered through a 0.22 μm membrane, and analyzed by radio-HPLC.

Additionally, cellular uptake studies were performed using the AR42J cell line. Briefly, AR42J cells (5 × 10^4^ cells/well) were seeded in 24-well plates and incubated overnight. On the following day, the cells were incubated in parallel with the same batch of 37 kBq of [^68^Ga]Ga-DOTA-CCK_2_R-dimer, [^68^Ga]Ga-DOTA-CCK-66, or [^177^Lu]Lu-DOTA-CCK_2_R-dimer at 37 °C for 30, 60, and 120 min (n = 5/group). After incubation, the cells were washed with PBS and lysed with 1 M NaOH, and the radioactivity was measured using a γ-counter. To evaluate binding specificity, blocking experiments were conducted in parallel using a 400-fold excess of the lead compound at the 1-h time point. The detailed procedures, including AR42J cell culture, are described in **Supplementary Methods Section S2**.

### *In Vivo* Experiments

To evaluate the clearance rates of [^68^Ga]Ga-DOTA-CCK_2_R-dimer and [^68^Ga]Ga-DOTA-CCK-66 from the blood, a pharmacokinetic study was conducted in male KM mice (4-6 weeks old, 18-20 g, n = 5/group). Each mouse received 3.7 MBq of radiotracer via tail vein injection, corresponding to approximately 120 pmol of [^68^Ga]Ga-DOTA-CCK_2_R-dimer or 210 pmol of [^68^Ga]Ga-DOTA-CCK-66. Blood samples were collected from the tail vein into preweighed capillary tubes at 1, 3, 5, 10, 15, 30, 60, 90, 120, and 150 min post-injection. The radioactivity of the blood samples was measured using a γ-counter, and the percent of injected dose per gram of tissue (%ID/g) was calculated. Additionally, we assessed the *in vivo* stability of [^177^Lu]Lu-DOTA-CCK_2_R-dimer. Mice were intravenously injected with 7.4 MBq (≈ 240 pmol) of [^177^Lu]Lu-DOTA-CCK_2_R-dimer (dissolved in 200 μL of saline; n = 3/group). Urine samples were collected at 1, 4, and 24 hours post-injection, then diluted, filtered through a 0.22 μm membrane, and analyzed by radio-HPLC.

### Micro-PET/CT Imaging

For the micro-positron emission tomography/computed tomography (microPET/CT) imaging study, BALB/c nude mice bearing AR42J tumors (detailed in **Supplementary Methods Section S3**: Mouse Models) were intravenously injected with 3.7 MBq of [^68^Ga]Ga-DOTA-CCK_2_R-dimer (≈ 120 pmol) or [^68^Ga]Ga-DOTA-CCK-66 (≈ 210 pmol) (dissolved in 200 μL of saline; n = 3/group), and microPET/CT imaging was performed at 30, 60, and 120 min post-injection. The mice were anesthetized via inhalation of an isoflurane mixture approximately 5 min before each PET/CT acquisition began. To confirm tumor-specific radiopharmaceutical uptake, blocking experiments were conducted at the 1-h time point of imaging by coinjecting the mice with the radiopharmaceutical and a 400-fold excess of the lead compound.

### Biodistribution of ^68^Ga-Labeled Compounds

To complement the imaging studies, biodistribution experiments were performed. AR42J tumor-bearing mice were intravenously injected with 3.7 MBq of [^68^Ga]Ga-DOTA-CCK_2_R-dimer (≈ 120 pmol) or [^68^Ga]Ga-DOTA-CCK-66 (≈ 210 pmol) (dissolved in 200 μL of saline; n = 3/group). The mice were sacrificed at 30, 60, and 120 min post-injection, and the tissues of interest were excised, weighed, and analyzed for radioactivity using a γ-counter. For blocking studies, a 400-fold excess of the lead compound was co-administered, and the mice were sacrificed at 1 h post-injection.

### Autoradiography with H&E Staining and Immunofluorescence

To analyze the distribution of the radiotracer, autoradiography, hematoxylin-eosin (H&E) staining, and CCK_2_R immunofluorescence (IF) analyses were performed on the same tissue sections. For autoradiography, the selected sections were placed on an imaging plate, and after 2.5 h of exposure, the plate was analyzed to visualize the radioactive signals from [^68^Ga]Ga-DOTA-CCK_2_R-dimer. The same sections were subsequently subjected to H&E staining and CCK_2_R IF staining to further evaluate tissue morphology and receptor expression further.

The CCK_2_R IF analysis (4A5, Santa Cruz Biotechnology, USA) was conducted following a previously established protocol 12. Briefly, tissue sections were blocked with 5% BSA at room temperature for 1 h and then incubated overnight at 4 °C with a recombinant mouse monoclonal anti-CCK_2_R antibody (1:500 dilution). After washing, the slides were incubated with an Alexa Fluor^®^ 594-conjugated goat anti-mouse IgG secondary antibody (Thermo Fisher Scientific, cat# A-11032, 1:1000 dilution) at room temperature for 1 h. Nuclei were counterstained with DAPI (1 μg/mL), and fluorescence images were acquired using a confocal microscope (Leica SP8).

### Micro-SPECT/CT Imaging

For the micro-single-photon emission computed tomography/computed tomography (microSPECT/CT) imaging study, AR42J tumor-bearing mice were intravenously injected with 7.4 MBq (≈ 240 pmol) of [^177^Lu]Lu-DOTA-CCK_2_R-dimer (dissolved in 200 μL of saline; n = 3/group), and SPECT/CT imaging was conducted at 1, 4, 24, 48, 72, and 96 h post-injection. The mice were anesthetized via inhalation of an isoflurane mixture approximately 5 min prior to each imaging session. SPECT/CT image analysis was performed to verify the tumor-targeting specificity of the radiopharmaceutical using VivoQuant 2.5 software.

### Biodistribution of ^177^Lu-Labeled Compounds

To evaluate the biodistribution of [^177^Lu]Lu-DOTA-CCK_2_R-dimer, AR42J tumor-bearing mice received intravenous (i.v.) injections of 3.7 MBq (≈ 120 pmol) of [^177^Lu]Lu-DOTA-CCK_2_R-dimer (dissolved in 200 μL of saline; n = 3/group), and Mice were sacrificed at 1, 4, 24, 48, 72, and 96 h post-injection. The tissues of interest were excised, weighed, and analyzed for radioactivity using a γ-counter.

### *In Vivo* Therapeutic Study

When the tumor volume reached approximately 100 mm^3^, the AR42J tumor-bearing mice were randomly divided into four treatment groups (n = 8/group). Group A (vehicle, Veh) received intraperitoneal (i.p.) injections of PBS; Group B (mTOR inhibitor, mTOR) was treated with 3 mg/kg everolimus (RAD001, MedChemExpress, Monmouth Junction, NJ, USA) via intraperitoneal (i.p.) injection every day for 10 consecutive days; Group C ([^177^Lu]Lu-DOTA-CCK_2_R-dimer, Lu) received a single i.v. injection of 18.5 MBq (≈ 600 pmol) of [^177^Lu]Lu-DOTA-CCK_2_R-dimer on day 5; and Group D (combination therapy, Combo) was treated with a combination of 18.5 MBq (≈ 600 pmol) of [^177^Lu]Lu-DOTA-CCK_2_R-dimer (administered via i.v. injection on day 5) and everolimus (RAD001, 3 mg/kg, i.p. daily for 10 days). This combination regimen was based on prior studies demonstrating the synergistic antitumor effects of combining [^177^Lu]Lu-PP-F11N (a radiopharmaceutical targeting CCK_2_R) with RAD001, and showed no observable toxicity 11, 13.

Tumor volumes and body weights were monitored every two days. The following humane endpoints were established as follows: tumor volume exceeding 1500 mm^3^, weight loss greater than 15%, or observable signs of pain or distress. Mice meeting one of these criteria were euthanized.

On day 12 post-treatment, the tumor tissues were processed and subjected to scRNA-seq and immunohistochemical (IHC) analysis (see **Supplementary Methods, Section S4**). To investigate the molecular mechanisms underlying the therapeutic effects of [^177^Lu]Lu-DOTA-CCK_2_R-dimer and RAD001, AR42J cells cultured *in vitro* were subjected to reactive oxygen species (ROS) detection, Western blotting, and RNA-sequencing (**Supplementary Methods, Section S5**). Toxicity studies were conducted when the mice reached one of the predefined endpoints (**Supplementary Methods, Section S6**).

### Statistical Analysis

All of the experimental data are presented as the means ± standard errors of the means (SEMs). Statistical analyses were performed using GraphPad Prism software (version 9.0, San Diego, USA). Unpaired t-tests were used to assess the significance of differences between groups, which was defined as *p* < 0.05. The median survival time was calculated using the Kaplan-Meier method, and the differences between groups were evaluated using the log-rank test.

## Results

### Molecular Docking Studies

**Figure 1:**
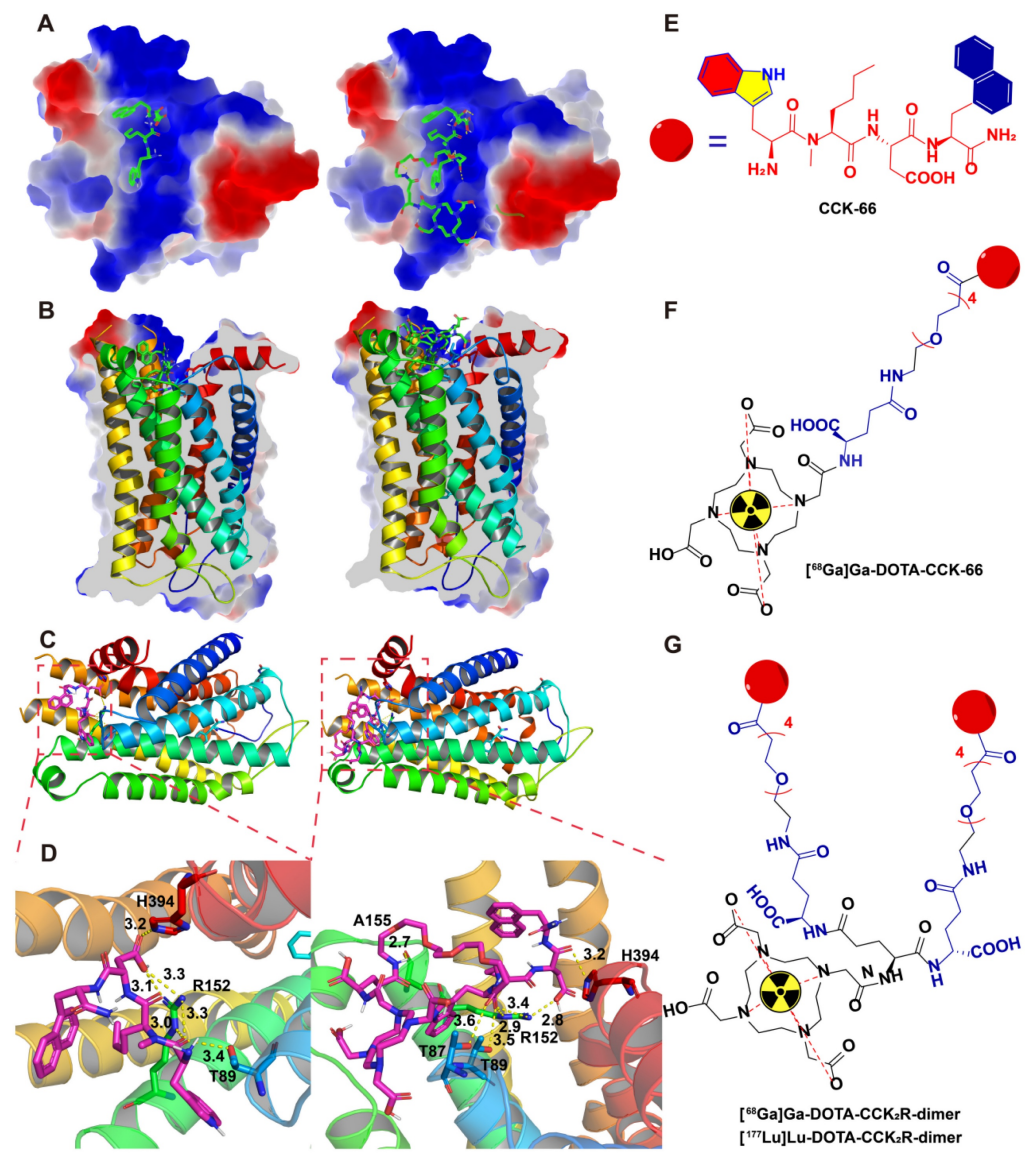
** Binding modes of CCK-66 and DOTA-CCK-66 to CCK_2_R (PDB ID: 7F8V).** (A) Co-crystal structures of CCK-66 (left) and DOTA-CCK-66 (right) bound to the binding pocket of CCK_2_R. (B) Cross-sectional views of the co-crystal structures of CCK-66 (left) and DOTA-CCK-66 (right) bound to binding pocket of CCK_2_R. (C) Binding modes of CCK-66 (left) and DOTA-CCK-66 (right) to CCK_2_R. (D) H-bond interactions between CCK-66 (Left) and DOTA-CCK-66 (right) and the residues of CCK_2_R. (E) Structures of CCK-66, (F) [^68^Ga]Ga-DOTA-CCK-66, (G) [^68^Ga]Ga-DOTA-CCK_2_R-dimer and [^177^Lu]Lu-DOTA-CCK_2_R-dimer.

To design multimeric probes with greater potential for imaging applications, it is necessary to understand the interactions among the lead compound and monomer with the CCK_2_R proteins. The high-resolution cryo-electron microscopy (EM) structure of the cholecystokinin receptor CCK_2_R in complex with gastrin-17 and Gi [PDB ID: 7F8V] provided insights into the binding mode. To elucidate the molecular basis underlying the affinity of the previously reported compound CCK-66 and its monomeric analog DOTA-CCK-66 for CCK_2_R, we analyzed their binding interactions in cocrystal structures (**Figure 1**). The surface electrostatic potential demonstrated that both ligands exhibit distinct binding orientations within the CCK_2_R binding pocket (**Figure 1A-B**). Detailed analysis revealed that both CCK-66 and DOTA-CCK-66 occupied the same hydrophobic pocket and engaged in complementary electrostatic interactions that aligned closely with critical transmembrane domains of the receptor (**Figure 1C**). Furthermore, CCK-66 formed hydrogen bonds with residues ARG-152, THR-89, and HIS-394, whereas DOTA-CCK-66 formed additional hydrogen bonding interactions with THR-87 and ALA-155, suggesting increased binding stability and specificity of DOTA-CCK-66 (**Figure 1D**).

On the basis of the aforementioned structural insights of CCK-66 (**Figure 1E**) and DOTA-CCK-66 bound to the receptor, we designed the dimeric radiotracer, DOTA-CCK_2_R-dimer, which incorporates either ^68^Ga or ^177^Lu radionuclides, and hypothesized that, compared with the monomer [^68^Ga]Ga-DOTA-CCK-66, receptor avidity would be improved with the dimer owing to multivalency effects (**Figure 1F-G**). These structural characterizations underscore the potential of [^68^Ga]Ga-DOTA-CCK_2_R-dimer and [^177^Lu]Lu-DOTA-CCK_2_R-dimer as promising molecular imaging tools and radiotherapeutic candidates to target CCK_2_R-positive tumors. Additionally, the structures of DOTA-CCK_2_R-dimer and DOTA-CCK-66 were confirmed via mass spectrometry (**Figure S1**).

### Radiolabeling

All radiolabeled products were determined to have radiochemical purities exceeding 98% by radio-HPLC, and therefore did not require further purification. The retention times of the labelled compounds closely matched those of the corresponding unlabeled peptides determined by UV-HPLC under identical chromatographic conditions: 13.89 min for the DOTA-CCK_2_R-dimer precursor, compared to 14.03 min and 14.02 min for the [^68^Ga]Ga- and [^177^Lu]Lu-labeled products, respectively; and 12.98 min for the DOTA-CCK-66 precursor, compared to 12.96 min for [^68^Ga]Ga-DOTA-CCK-66** (Figure S2)**. These results confirm that the radionuclides were incorporated into the compounds without significantly altering their molecular structures. The detailed analysis parameters are summarized in **Table S1**.

### [^68^Ga]Ga-DOTA-CCK_2_R-dimer Exhibits High Hydrophilicity and Stability and a Prolonged Circulation Time

[^68^Ga]Ga-DOTA-CCK_2_R-dimer and [^68^Ga]Ga-DOTA-CCK-66 showed similarly high hydrophilicity under physiological conditions, with Log D_7.4_ values of -3.01 ± 0.05 and -3.03 ± 0.03, respectively** (Figure S3)**.

Both radiotracers demonstrated excellent *in vitro* stability in PBS and human serum at 37 °C, with no demetallation or free radioisotope release detected by radio-HPLC after 180 min** (Figure S4)**.

Both radiotracers were rapidly distributed initially, with t_1/2α_ values of 0.63 min for [^68^Ga]Ga-DOTA-CCK_2_R-dimer and 0.67 min for [^68^Ga]Ga-DOTA-CCK-66. Regarding clearance, [^68^Ga]Ga-DOTA-CCK_2_R-dimer exhibited a t_1/2β_ of 46.60 min, compared with 11.80 min for [^68^Ga]Ga-DOTA-CCK-66, suggesting a prolonged circulation time of the dimer (**Figure S5**).

### Cellular Uptake and Receptor Specificity of [^68^Ga]-DOTA-CCK_2_R-dimer and [^177^Lu]-DOTA-CCK_2_R-dimer

*In vitro* uptake experiments conducted with AR42J cells demonstrated that [^68^Ga]Ga-DOTA-CCK_2_R-dimer exhibited significantly greater uptake at all time points than did [^68^Ga]Ga-DOTA-CCK-66 (0.5 h: 4.00 ± 0.21% vs. 3.00 ± 0.21%; 1 h: 4.50 ± 0.88% vs. 3.33 ± 0.54%; and 2 h: 4.33 ± 0.52% vs. 3.33 ± 0.52%; *p* < 0.05; **Figure S6**). Upon co-incubation with excess unlabeled ligand, the 1-h uptake decreased to 1.15 ± 0.26% and 0.87 ± 0.19%, respectively, further confirming the CCK_2_R specificity of both radiotracers.

To validate the receptor-mediated uptake of [^177^Lu]Lu-DOTA-CCK_2_R-dimer, a similar *in vitro* uptake experiment was performed with AR42J cells. The radiotracer showed consistent cellular uptake over time (0.5 h: 2.92 ± 0.42%; 1 h: 3.59 ± 0.60%; and 2 h: 3.46 ± 0.32%), which significantly decreased to 0.79 ± 0.25% (*p* < 0.001; **Figure S7**) following co-incubation with excess unlabeled ligand, further supporting its high binding specificity for CCK_2_R.

### Imaging and Biodistribution Studies Confirm the Superior Tumor-Targeting Capability of [^68^Ga]Ga-DOTA-CCK_2_R-dimer

**Figure 2:**
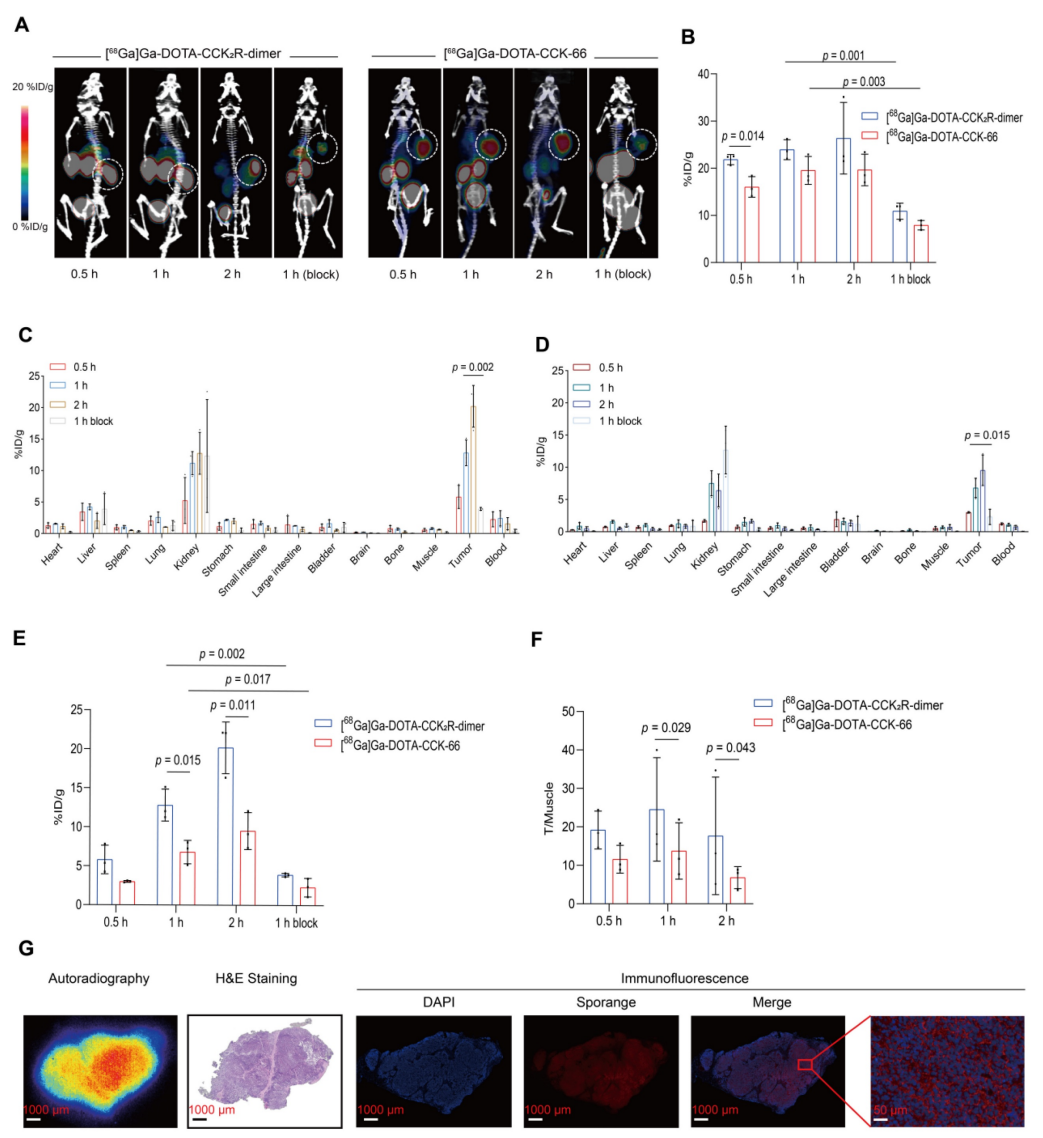
** [^68^Ga]Ga-DOTA-CCK_2_R-dimer exhibits superior tumor-targeting ability in imaging and biodistribution studies.** (A) MicroPET/CT images of AR42J tumor-bearing mice injected with [^68^Ga]Ga-DOTA-CCK_2_R-dimer (left) or [^68^Ga]Ga-DOTA-CCK-66 (right). Tumors are indicated by white dashed circles. Each treatment included a 1-h blocking condition with co-injection of excess unlabeled ligand to assess receptor specificity. Images were acquired at 0.5, 1, and 2 h postinjection (n = 3/group). The color scale represents %ID/g. (B) Comparison of [^68^Ga]Ga-DOTA-CCK_2_R-dimer and [^68^Ga]Ga-DOTA-CCK-66 tumor uptake in AR42J tumor-bearing mice on the basis of the microPET/CT images (n = 3). (C) *Ex vivo* biodistribution analysis of [^68^Ga]Ga-DOTA-CCK_2_R-dimer in AR42J tumor-bearing mice (n = 3). (D) *Ex vivo* biodistribution analysis of [^68^Ga]Ga-DOTA-CCK-66 in AR42J tumor-bearing mice (n = 3). (E) Comparison of [^68^Ga]Ga-DOTA-CCK_2_R-dimer and [^68^Ga]Ga-DOTA-CCK-66 tumor uptake on the basis of the *ex vivo* biodistribution analysis (n = 3). (F) Comparison of tumor-to-muscle (T/M) ratios of [^68^Ga]-Ga-DOTA-CCK_2_R-dimer and [^68^Ga]Ga-DOTA-CCK-66 on the basis of the *ex vivo* biodistribution data (n = 3). (G) Autoradiographical, histological, and immunofluorescence analysis of CCK_2_R expression and tracer uptake. Autoradiography images showing the intratumoral distribution of [^68^Ga]Ga-DOTA-CCK_2_R-dimer; the regions of high tracer accumulation are highlighted. Hematoxylin and eosin (H&E) staining revealed the histological features of AR42J tumor sections, providing a morphological context for tracer localization. Representative immunofluorescence (IF) staining images confirming high CCK_2_R expression in AR42J tumors; CCK_2_R expression was spatially correlated with the areas of tracer accumulation observed via autoradiography. Scale bars are indicated in each image.

To comprehensively assess the *in vivo* imaging performance of [^68^Ga]Ga-DOTA-CCK_2_R-dimer, we conducted micro-PET/CT imaging studies in BALB/c nude mice bearing AR42J tumors. As shown in **Figure 2A**, [^68^Ga]Ga-DOTA-CCK_2_R-dimer exhibited rapid tumor uptake within 30 min, achieving a discernible tumor accumulation of 22.63 ± 0.32 %ID/g. Radiotracer uptake steadily increased to 23.93 ± 2.12 %ID/g at 60 min and further to 26.13 ± 6.21 %ID/g at 120 min, demonstrating sustained retention at the tumor site. In the blocking group, co-injection with the unlabeled ligand significantly reduced tumor uptake from 23.93 ± 2.12 %ID/g to 10.90 ± 1.73 %ID/g at the 1-hour time point, confirming receptor-specific binding (*p* = 0.001). Notably, [^68^Ga]Ga-DOTA-CCK-66 also demonstrated rapid tumor uptake within 30 min, with uptake rates of 16.03 ± 2.18 %ID/g, 19.53 ± 2.95 %ID/g, and 19.63 ± 3.35 %ID/g at 30, 60, and 120 min, respectively. Compared with the uptake rates of [^68^Ga]Ga-DOTA-CCK_2_R-dimer, those of [^68^Ga]Ga-DOTA-CCK-66 were lower at all time points, with a particularly significant difference at the 30 min time point (*p* = 0.014; **Figure 2B**). Blocking studies confirmed the receptor specificity of [^68^Ga]Ga-DOTA-CCK-66, as its tumor uptake at 1 h decreased significantly from 19.53 ± 2.95 %ID/g to 7.90 ± 1.35 %ID/g after co-injection with the unlabeled ligand (*p* = 0.003).

To validate the *in vivo* tumor-targeting capability of [^68^Ga]Ga-DOTA-CCK_2_R-dimer, we conducted *ex vivo* biodistribution studies of treated AR42J tumor-bearing mice (**Figure 2C-D**). At 30 min post-injection, the tumor uptake rates of [^68^Ga]Ga-DOTA-CCK_2_R-dimer and [^68^Ga]Ga-DOTA-CCK-66 were 5.81 ± 1.83 %ID/g and 3.00 ± 0.63 %ID/g (**Table S2**), respectively, indicating substantial tumor accumulation. Over time, tumor uptake steadily increased, with [^68^Ga]Ga-DOTA-CCK_2_R-dimer demonstrating significantly greater tumor uptake than [^68^Ga]Ga-DOTA-CCK-66 did at both 1 h (12.80 ± 2.05 vs. 6.79 ± 1.50; *p* = 0.015) and 2 h (20.19 ± 3.31 vs. 9.53 ± 2.38; *p* = 0.011) (**Figure 2E**); notably, blocking with excess of unlabeled ligand significantly reduced the uptake of [^68^Ga]Ga-DOTA-CCK_2_R-dimer after 1 h to 3.91 ± 1.23 (*p* = 0.002) and that of [^68^Ga]Ga-DOTA-CCK-66 to 2.35 ± 1.18 (*p* = 0.017), further confirming receptor-specific accumulation (**Figure 2E**). Determination of the tumor-to-muscle (T/M) ratios revealed that [^68^Ga]Ga-DOTA-CCK_2_R-dimer presented significantly higher values than did [^68^Ga]Ga-DOTA-CCK-66 at both 1 h (16.54 ± 2.99 vs. 10.25 ± 1.41; *p* = 0.029) and 2 h post-injection (33.03 ± 7.91 vs. 15.20 ± 6.94; *p* = 0.043; **Figure 2F**). These results indicate that [^68^Ga]Ga-DOTA-CCK_2_R-dimer exhibits sustained tumor uptake and favorable tumor-to-background contrast. Both radiotracers showed the greatest accumulation in the kidneys, which is consistent with the PET imaging findings, suggesting that renal clearance is the primary excretion route. Uptake by other nontarget organs, including the heart, stomach, and intestines, remained low at all time points, further supporting the overall specificity of tumor targeting. The specific tumor-to-background ratios at each time point are provided in **Table S3**.

### [^68^Ga]Ga-DOTA-CCK_2_R-dimer Uptake Reflects CCK_2_R Expression

To investigate the spatial relationship between radiotracer [^68^Ga]Ga-DOTA-CCK_2_R-dimer uptake and CCK_2_R target protein expression, autoradiography, H&E staining, and IF analyses were performed on tumor sections, as illustrated in **Figure 2G**. Autoradiography revealed significant radiotracer accumulation in tumor regions, the distribution of which closely matched the morphological characteristics of the tumors observed in the H&E-stained sections. IF further confirmed the specific localization of CCK_2_R, demonstrating robust expression predominantly in tumor tissues. The observed overlap of [^68^Ga]Ga-DOTA-CCK_2_R-dimer and CCK_2_R receptor expression supports the specificity of this radiotracer.

### Biodistribution and Imaging Studies Support Sustained Tumor Accumulation of [^177^Lu]Lu-DOTA-CCK_2_R-dimer in Tumors

The radiotracer demonstrated excellent stability, with radiochemical purities above 99.3% in PBS and 99.0% in human serum after 96 h of incubation. *In vivo* stability was confirmed by urinalysis, which revealed intact tracer purities of 99.6%, 99.8%, and 98.0% at 1, 4, and 24 h post-injection, respectively (**Figure S8**).

**Figure 3:**
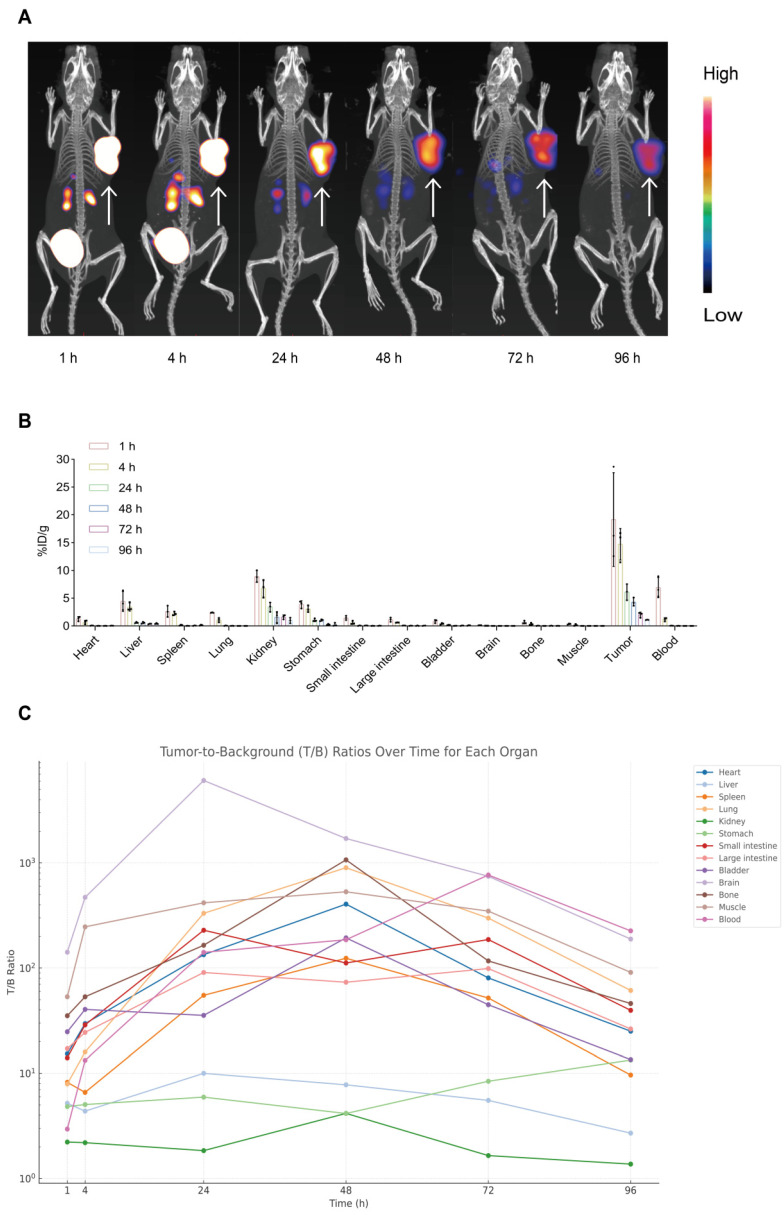
** Longitudinal micro-SPECT imaging, biodistribution, and tumor-to-background ratio analyses of [^177^Lu]Lu-DOTA-CCK_2_R-dimer in AR42J tumor-bearing mice.** (A) Micro-SPECT/CT images of AR42J tumor-bearing mice at 1, 4, 24, 48, 72, and 96 h after injection of [^177^Lu]Lu-DOTA-CCK_2_R-dimer. The arrows indicate the tumor locations. (B) Time-dependent *ex vivo* biodistribution of [^177^Lu]Lu-DOTA-CCK_2_R-dimer in AR42J tumor-bearing mice at 1, 4, 24, 48, 72, and 96 h post-injection (n = 3 per time point; means ± SDs). (C) Tumor-to-background (T/B) ratios were measured *ex vivo* at 1, 4, 24, 48, 72, and 96 h post-injection. Each curve represents the dynamic T/B profile of an individual organ, illustrating organ-specific tracer clearance and background signal kinetics over time (n = 3 per time point; means ± SDs).

To assess tumor-targeting performance, serial microSPECT/CT imaging of AR42J tumor-bearing mice was conducted at 1, 4, 24, 48, 72, and 96 h post-injection. As shown in **Figure 3A**, the tracer accumulated in tumors within 1 h and remained clearly detectable for up to 96 h. This prolonged tumor localization suggests high affinity for the target receptor (CCK_2_R) and excellent tumor retention, demonstrating the effectiveness of [^177^Lu]Lu-DOTA-CCK_2_R-dimer for *in vivo* imaging applications and providing support for targeted radionuclide therapy.

We conducted *ex vivo* biodistribution studies with AR42J tumor-bearing mice to further evaluate the tumor-targeting ability of [^177^Lu]Lu-DOTA-CCK_2_R-dimer *in vivo*. The distribution of the radiotracer was analyzed at 1, 4, 24, 48, 72, and 96 h postinjection (**Figure 3B**). At 1 h, the radiotracer showed significant accumulation in the primary tumor (19.17 ± 8.43 %ID/g) and remained detectable at all subsequent time points. Initial renal uptake (8.92 ± 1.05 %ID/g) and hepatic uptake (4.44 ± 1.80 %ID/g) indicated predominant renal clearance with partial hepatobiliary metabolism. Minimal uptake was observed in nontarget tissues such as the heart, brain, and muscle, supporting the tumor specificity of [^177^Lu]Lu-DOTA-CCK_2_R-dimer (**Table S4**).

Notably, the tumor-to-blood ratio increased from 2.95 ± 1.50 at 1 h to 185.72 ± 14.28 at 48 h and reached 225.66 ± 40.93 at 96 h, indicating progressive clearance from circulation and increased tumor selectivity over time. Similarly, the tumor-to-kidney ratio increased from 2.22 ± 1.01 at 1 h to 4.16 ± 3.43 at 48 h despite its renal clearance, and the tumor-to-stomach ratio increased from 4.83 ± 1.46 at 1 h to 13.36 ± 6.42 at 96 h, suggesting minimal interference of physiologically expressed CCK_2_R. These results quantitatively support the high tumor specificity and prolonged retention of [^177^Lu]Lu-DOTA-CCK_2_R-dimer *in vivo* (**Figure 3C, Table S5**).

### Synergistic Efficacy and Safety of [^177^Lu]Lu-DOTA-CCK_2_R-dimer and mTOR Inhibitor Combination Therapy

**Figure 4:**
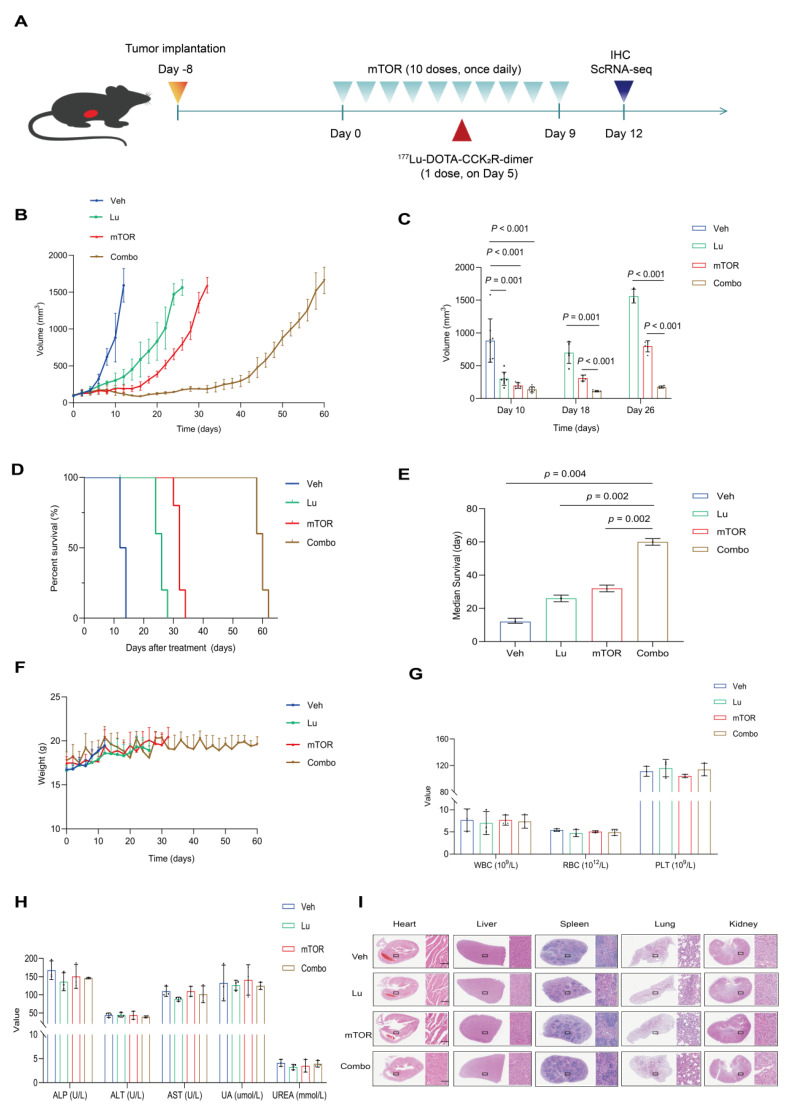
** Evaluation of the antitumor therapeutic efficacy of [^177^Lu]Lu-DOTA-CCK_2_R-dimer combined with an mTOR inhibitor in AR42J tumor-bearing mice.** (A) Schematic of the AR42J tumor-bearing mice experimental treatment protocol (n = 8/group). The studies included single agent treatments with an mTOR inhibitor (mTOR) or [^177^Lu]Lu-DOTA-CCK_2_R-dimer (Lu) and a combination therapy (Combo) group. (B) Tumor growth curves depicting tumor progression each of the four groups of AR42J tumor-bearing mice: vehicle (untreated) and three treatment groups (each agent alone and combination therapy). The data are presented as the mean tumor volumes ± SDs. (C) Bar graph illustrating the tumor volumes (means ± SDs) measured on days 10, 18, and 26 after treatment initiation, demonstrating the significant tumor growth inhibition in the combination therapy group. (D) Kaplan‒Meier survival curves of each of the four groups of AR42J tumor-bearing mice, indicating the significantly prolonged survival in the combination therapy group. (E) Comparison of the median survival time of the four groups, highlighting the significantly prolonged survival in the combination therapy group (*p* < 0.05). (F) Body weight changes in each of the four groups of AR42J tumor-bearing mice throughout the treatment period showing that no significant weight loss occurred. (G) Hematological analysis showing the white blood cell (WBC), red blood cell (RBC), and platelet (PLT) counts across the four groups. (H) Serum biochemical analysis assessing hepatic and renal function, including alkaline phosphatase (ALP), alanine aminotransferase (ALT), aspartate aminotransferase (AST), uric acid (UA), and urea (UREA) levels, across the four groups. (I) Histopathological evaluation of major organs (heart, liver, spleen, lungs, and kidneys) in the vehicle and treatment groups via H&E staining (n = 3/group). Scale bar = 100 µm. All images were acquired at 20× magnification and share the same scale.

To investigate whether mTOR inhibition enhances the therapeutic efficacy of [^177^Lu]Lu-DOTA-CCK_2_R-dimer, we evaluated their combination in an AR42J tumor-bearing mouse model. The experimental treatment scheme is shown in **Figure 4A**. The therapeutic effects were assessed on the basis of tumor growth, survival time, and histopathological changes at the specific treatment time points. Both [^177^Lu]Lu-DOTA-CCK_2_R-dimer and mTOR inhibitor monotherapy and their combination effectively inhibited tumor growth (**Figure 4B**). On day 10, all the mice were still alive, and the average tumor volume of the vehicle group was 883.09 ± 331.18 mm³. In contrast, the average tumor volumes of the [^177^Lu]Lu-DOTA-CCK_2_R-dimer, mTOR inhibitor, and combined treatment groups were significantly smaller (*p* < 0.05) at 302.72 ± 100.12 mm³, 197.63 ± 40.92 mm³, and 139.10 ± 39.86 mm³, respectively. Thus, combination treatment induced significantly greater tumor growth inhibition than either [^177^Lu]Lu-DOTA-CCK_2_R-dimer or the mTOR inhibitor alone. On days 18 and 26, the tumor size in the combination treatment group remained significantly lower than that in either monotherapy group (*p* < 0.05) (**Figure 4C**).

Compared with vehicle treatment, all the drug treatments significantly improved survival rates, as shown by the Kaplan-Meier survival curves (**Figure 4D**). The median survival time of the control group was 12 days, while those of the [^177^Lu]Lu-DOTA-CCK_2_R-dimer, mTOR inhibitor, and combination treatment groups were extended to 26, 32, and 60 days, respectively. **Figure 4E** shows a statistical comparison of the median survival times among the four groups, demonstrating that, compared with single agent treatment, combination treatment significantly prolonged the survival of these tumor-bearing mice (*p* < 0.05). These findings highlight the synergistic effect of combining [^177^Lu]Lu-DOTA-CCK_2_R-dimer with mTOR inhibition.

Throughout the treatment period, all the groups presented a continuous increase in body weight, and no significant weight loss was observed, suggesting that the treatments did not induce systemic toxicity (**Figure 4F**). Routine blood analysis revealed no significant differences in red blood cell (RBC), white blood cell (WBC), or platelet (PLT) counts between the treatment groups and the vehicle group (**Figure 4G**). Similarly, biochemical analysis revealed no notable deviations, with all treatment groups displaying values of key hepatic and renal function markers, including alkaline phosphatase (ALP), alanine aminotransferase (ALT), aspartate aminotransferase (AST), uric acid (UA), and urea (UREA), that were comparable to those in to the vehicle group (**Figure 4H**). Furthermore, histopathological analysis revealed no pathological abnormalities in any of the animals, indicating no acute organ toxicity (**Figure 4I**). The specific values of the routine blood analysis and biochemical parameters are provided in **Table S6** and **Table S7**.

Collectively, these results suggest that combination therapy with an mTOR inhibitor and [^177^Lu]Lu-DOTA-CCK_2_R-dimer not only outperforms each single agent treatment in terms of therapeutic efficacy but also exhibits an acceptable safety profile, with no significant side effects or organ toxicity observed.

### The [^177^Lu]Lu-DOTA-CCK_2_R-dimer Selectively Eliminates Highly Proliferative and Poorly Differentiated Tumor Cell Populations

**Figure 5:**
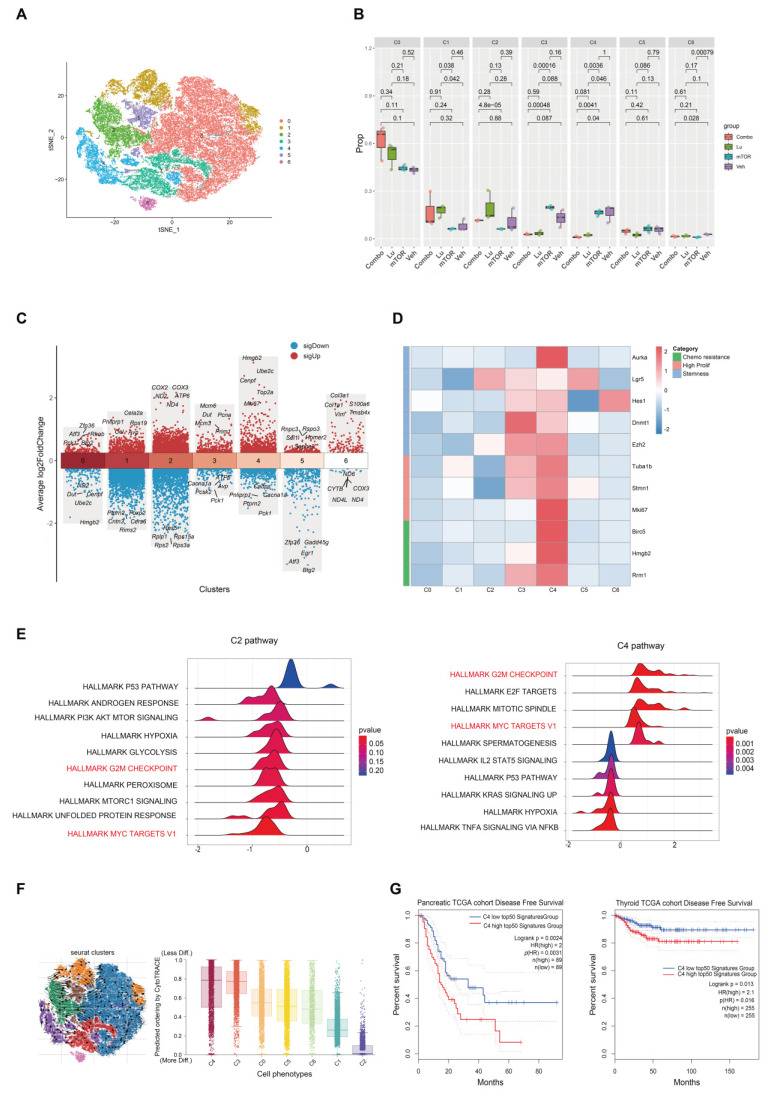
** Single-cell RNA sequencing reveals tumor cell heterogeneity across different treatment groups.** (A) t-SNE clustering analysis identified seven distinct tumor cell populations on the basis of their transcriptomic profiles. (B) Relative proportions of the cell populations across treatment groups. Compared with those in the Veh and mTOR groups, the proportions of the C3 and C4 populations in the Lu and Combo groups were significantly lower, with a particularly pronounced reduction in C4 cells (*p* < 0.05). (C) Differential gene expression analysis revealed elevated expression of DNA replication-associated genes (Mcm6, Mcm3, Pcna, Dut, and Prim1) in C3 cells, whereas C4 cells presented high expression of proliferation- and mitosis-related genes (Hmgb2, Ube2c, Cenpf, Top2a, and Mki67). (D) Heatmap analysis revealed significant enrichment of chemotherapy resistance-associated genes (Birc5, Hmgb2, and Rrm1), proliferation-related genes (Tuba1b, Stmn1, and Mki67), and stemness markers (Aurka, Lgr5, Hes1, Dnmt1, and Ezh2) in the C3 and C4 populations. (E) Gene set enrichment analysis (GSEA) revealed that the HALLMARK MYC TARGETS V1 and HALLMARK G2M CHECKPOINT pathways were significantly enriched in the genes differentially expressed in C4 cells, indicating their role in cell cycle progression and robust proliferative capacity. (F) Pseudotime trajectory analysis revealed that C3 and C4 cells were positioned in the early differentiation stage, whereas C1 and C2 cells were located at later differentiation stages, suggesting that the C3 and C4 populations exhibit stem-like characteristics. (G) TCGA survival analysis demonstrated that the increased expression of the top 50 C4-associated genes was significantly correlated with shortened disease-free survival (DFS) in pancreatic and thyroid cancer patients (*p* < 0.05), whereas low expression of these genes did not result in a similar association.

To investigate the therapeutic mechanism of [^177^Lu]Lu-DOTA-CCK_2_R-dimer, we performed scRNA-seq analysis on tumor samples from AR42J tumor-bearing mice in the four experimental groups (Veh, mTOR, Lu, and Combo). T-distributed stochastic neighbor embedding (t-SNE) clustering analysis revealed seven distinct cell populations (**Figure 5A**) that were evenly distributed across the different treatment groups, indicating that there was no significant batch effect (**Figure S9**). Further analysis revealed that the proportions of the C1 and C2 cell populations was lower in the Veh and mTOR groups, whereas the proportions of the C3 and C4 cell population was lower in the Lu and Combo groups (**Figure 5B**). Notably, the proportion of C3 cells was sinificantly lower in the Lu group than in the mTOR group (*p* < 0.05), and the proportion of C4 cells was significantly lower in the Lu and Combo groups than in the Veh and mTOR groups (*p* < 0.05). These findings suggest that [^177^Lu]Lu-DOTA-CCK_2_R-dimer primarily targets C3 and C4 ell populations and selectively eliminates tumor cells within these subgroups.

To further characterize the biological properties of each cell population, we analyzed the gene expression patterns using Seurat. The C3 cell population presented significantly increased expression of Mcm6, Mcm3, Pcna, Dut, and Prim1, all of which are involved in DNA replication and repair 14-17, suggesting that C3 cells possess high DNA replication and proliferative capacities.

The C4 cell population showed high expression of Hmgb2, Ube2c, Cenpf, Top2a, and Mki67, among which Hmgb2 and Ube2c are associated with tumor cell proliferation and drug resistance 18, 19, Cenpf and Top2a are key regulators of mitosis 20, 21, and Mki67 serves as a core marker of cell proliferation 22, Together, these data indicate that C4 cells exhibit robust proliferative activity (**Figure 5C**). In contrast, C1 and C2 cells lacked enrichment of canonical proliferation markers such as Mki67 and Pcna. Additionally, the C1 cell population exhibited high expression of Pnliprp1, Cela2a, Rps19, Cel, and Avp, suggesting a certain degree of pancreatic tissue specificity and the potential to exhibit neuroendocrine-like characteristics 23-25. Moreover, the C2 cell population presented high expression of the COX2, COX3, ND2, ATP6, and ND4 genes, which are all associated with mitochondrial electron transport chain function and oxidative phosphorylation (OXPHOS) 26-28 (**Figure 5C**). Additional analyses conducted to further investigate tumor cell heterogeneity (**Figure 5D-G**), and described in the Discussion section. These earlier analyses indicate that the C4 cell population may be a key subpopulation influencing the progression of tumor malignancy and drug resistance.

### mTOR Inhibitor Treatment Increases [^177^Lu]Lu-DOTA-CCK_2_R-dimer-Induced Oxidative Stress by Suppressing the GSH Metabolic Detoxification Pathway

**Figure 6:**
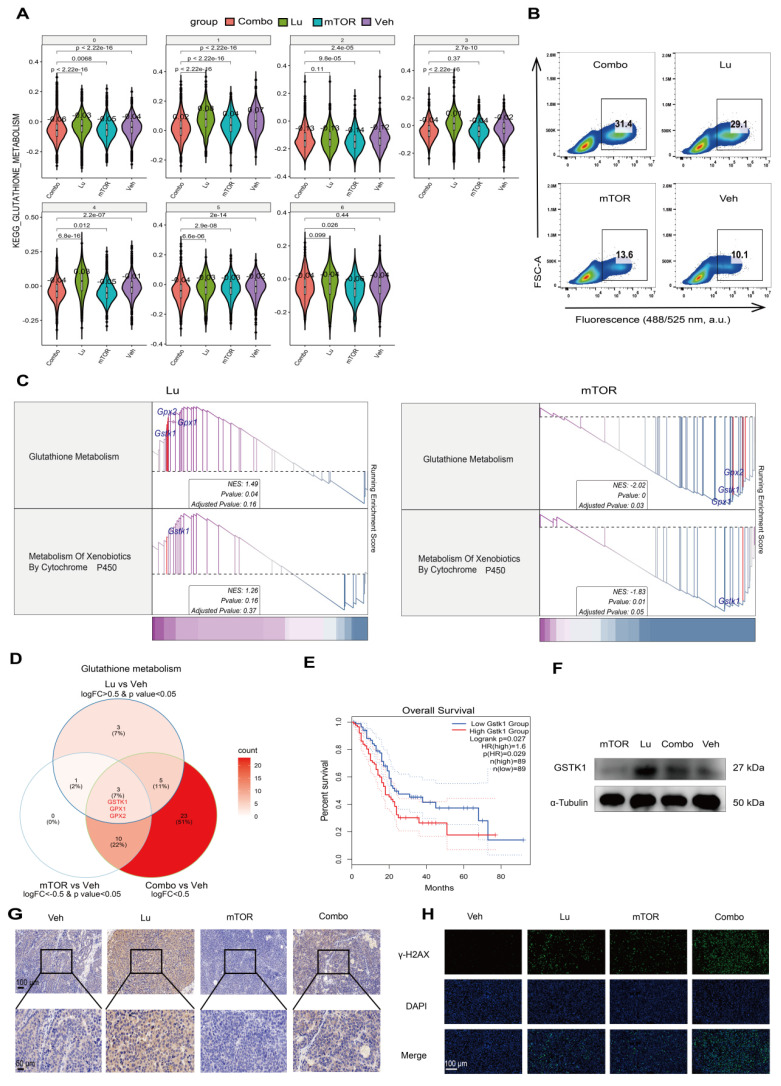
** mTOR inhibition increases [^177^Lu]Lu-DOTA-CCK_2_R-dimer-induced oxidative stress by suppressing glutathione metabolism.** (A) Violin plots depicting the expression levels of glutathione metabolism-related genes across different treatment groups (Veh, mTOR, Lu, and Combo) on the basis of the scRNA-seq data. The Lu group exhibited significant activation of glutathione metabolism, whereas the mTOR group presented significant suppression of this pathway. (B) Flow cytometry analysis of intracellular reactive oxygen species (ROS) levels in AR42J cells treated with [^177^Lu]Lu-DOTA-CCK_2_R-dimer (Lu), the mTOR inhibitor (mTOR), combination therapy (Combo), or vehicle (Veh). The results revealed a discernible increase in ROS levels in both the Lu-treated and combination therapy groups relative to the vehicle control and mTOR inhibitor groups, suggesting that oxidative stress was activated under these treatment conditions. (C) Gene set enrichment analysis (GSEA) of glutathione metabolism and cytochrome P450-mediated xenobiotic detoxification pathways in the Lu and mTOR treatment groups. The Lu group presented activation of these detoxification pathways, whereas the mTOR group presented significant suppression of these pathways. (D) Venn diagram illustrating the overlap of differentially expressed genes (DEGs) involved in glutathione metabolism in the Lu vs. Veh, mTOR vs. Veh, and Combo vs. Veh comparisons. GSTK1, GPX1, and GPX2 were identified as key regulatory genes within this pathway. (E) Kaplan‒Meier survival analysis of the TCGA pancreatic cancer (PAAD) dataset. Patients with high GSTK1 expression presented significantly shorter overall survival (OS) than did those with low GSTK1 expression (*p* = 0.027). (F) Western blot analysis of GSTK1 expression in AR42J cells following Lu, mTOR, Combo, or Veh treatment. GSTK1 was upregulated in the Lu group but downregulated in the mTOR and Combo groups, which is consistent with the RNA-seq results. (G) Immunohistochemical (IHC) staining of GSTK1 in tumor tissues from the Veh, Lu, mTOR, and Combo treatment groups. GSTK1 expression was notably higher in the Lu group but lower in the mTOR and Combo groups. Scale bars: upper panels, 100 µm; lower panels, 50 µm. (H) Immunofluorescence (IF) staining of γ-H2AX in tumor sections from different treatment groups. The γ-H2AX signal was strongest in the Combo group, indicating the greatest DNA damage. Scale bar: 100 µm.

To investigate the effect of the mTOR inhibitor RAD001 on the therapeutic efficacy of [^177^Lu]Lu-DOTA-CCK_2_R-dimer, we calculated the average expression levels of the mTOR-related gene sets on the basis of the scRNA-seq data. Compared with the Lu and Veh groups, the mTOR group exhibited suppression of mTOR downstream signaling pathways in all seven cell populations (**Figure S11**), suggesting that unlike Lu treatment, mTOR inhibitor treatment exerts tumor killing effects uniformly across different subpopulations by suppressing downstream signaling. Further pathway enrichment analysis revealed opposing trends in the glutathione metabolism pathway between the Lu group and the mTOR group, where the C3 and C4 cell subpopulations were significantly increased in the Lu group but decreased in the mTOR group (**Figure 6A**).

Previous studies have shown that radiotherapy can induce ROS production, while the glutathione (GSH)-mediated detoxification pathway plays a critical roles in regulating ROS levels and radiosensitivity 29, 30. However, because there are multiple variables in *in vivo* experiments, it is challenging to directly monitor the responses of relevant pathways. Therefore, to assess the direct effects of [^177^Lu]Lu-DOTA-CCK_2_R-dimer and the mTOR inhibitor on oxidative stress more precisely, we conducted a short-term transient intervention experiment in the AR42J cell model. In this experiment, the cells were treated with [^177^Lu]Lu-DOTA-CCK_2_R-dimer, RAD001, a combination of both treatments (Combo), or a vehicle control (Veh) for 24 h. To quantitatively assess oxidative stress levels, we measured intracellular ROS production using flow cytometry, which allowed us to further investigate the effects of different treatment strategies on cellular redox homeostasis. Compared with those in the Veh and mTOR groups, the ROS levels in the Lu and Combo groups tended to increase (**Figure 6B**).

Further RNA-seq analysis of the in vitro cell samples revealed that, compared with the Veh group, the Lu group presented significant activation of the glutathione metabolism pathway and xenobiotic detoxification by the cytochrome P450 pathway, whereas these pathways were significantly suppressed in the mTOR group (**Figure 6C**). Notably, both of these metabolic pathways are closely associated with radiotherapy-related detoxification mechanisms. The glutathione metabolism pathway plays a critical role in scavenging ROS, repairing oxidative damage, and protecting cells, whereas the cytochrome P450-mediated detoxification pathway is both involved in the metabolism of drugs and toxic compounds and plays an important role in oxidative stress regulation 31, 32. These findings suggest that [^177^Lu]Lu-DOTA-CCK_2_R-dimer may alleviate the oxidative stress burden in tumor cells by activating radiotherapy-related detoxification pathways, whereas the addition of an mTOR inhibitor may increase therapeutic efficacy by suppressing these pathways, thus increasing the susceptibility of tumor cells to oxidative damage.

To further elucidate the key regulatory molecules involved in the differential modulation of GSH-related oxidative stress, we performed gene screening and pathway analyses. Glutathione S-transferase kappa 1 (GSTK1) emerged as a shared regulator of both the glutathione metabolism and cytochrome P450 detoxification pathways, as it was upregulated by [^177^Lu]Lu-DOTA-CCK_2_R-dimer treatment but downregulated upon mTOR inhibition (**Figure 6D**). Notably, elevated GSTK1 expression has been associated with poorer prognosis in pancreatic cancer patients and has been strongly positively correlated with the C4 malignant subpopulation signature (**Figure 6E-F**). These findings highlight GSTK1 as a potential mediator of oxidative stress responses during therapy. Mechanistic implications are further elaborated in the Discussion section.

## Discussion

CCK_2_R is highly expressed in neuroendocrine tumors, making it a significant target in oncology. Building on previous studies demonstrating the favorable targeting and stability of monomeric CCK_2_R radiotracers, we designed and synthesized an innovative dimeric radiotracer, [^68^Ga]Ga/[^177^Lu]Lu-DOTA-CCK2R-dimer, using a conformation-unrestricted strategy to achieve favorable targeting and *in vivo* stability. Our results demonstrate that this dimeric radiotracer has high *in vitro* and *in vivo* stability, displays favorable imaging contrast, and achieves notable tumor retention as evidenced by serial imaging and biodistribution analyses. Furthermore, the mTOR inhibitor RAD001 increases the therapeutic efficacy of [^177^Lu]Lu-DOTA-CCK_2_R-dimer by modulating oxidative stress and glutathione metabolism. Importantly, this study demonstrates that this radiotherapeutic agent can selectively target and eliminate poorly differentiated, highly proliferative tumor cell subpopulations. Additionally, we reveal that an mTOR inhibitor can increase tumor cell sensitivity to radiation-induced damage by downregulating GSTK1 expression, thereby weakening glutathione-mediated ROS clearance. These findings offer a novel strategy for CCK_2_R-targeted radiotherapy.

Recent developments in CCK_2_R-targeted probe design have focused on improving *in vivo* stability and reducing off-target organ uptake. Early probes such as MGS5 introduced modifications to enhance tumor affinity and lower renal accumulation; this structure was further refined (DOTA-CCK-66) by optimizing the N-terminal γ-D-Glu sequence and using PEG linkers to improve metabolic performance 3, 4. More recently, efforts have also shifted toward modulating peptide conformation. Studies have explored the use of β-hairpin foldamer motifs to improve structural rigidity and biological activity. Moreover, the folded peptides exhibit increased binding affinity, complex structural characteristics, and improved tumor targeting 7, 33, 34. Collectively, these strategies have contributed to the ongoing optimization of CCK_2_R-targeted radiopharmaceuticals. This study introduced two key structural optimizations based on existing CCK_2_R-targeted radiotracers: the multivalency effect and the design of a conformation-unrestricted dimeric structure.

One of the key findings of this study is that designing a dimeric CCK_2_R-targeted radiotracer significantly enhances radiotracer uptake by tumor tissue through a multivalency effects. In our experiments, the dimeric construct, [^68^Ga]Ga-DOTA-CCK_2_R-dimer consistently exhibited greater uptake by AR42J tumors than its monomeric counterpart did, with this trend remaining consistent at different time points (30 min to 2 h p.i.). Notably, to maintain a consistent amount of the precursor during radiolabeling, the molar activity of the dimer was relatively high, which may have partially influenced its tumor uptake. Therefore, although the increased uptake observed with the dimer is consistent with the expected multivalency effect and aligns with findings of other multimeric tracers targeting fibroblast activation protein (FAP) and arginine-glycine-aspartic acid (RGD) 35, 36, it should be interpreted in light of these experimental conditions. Future studies could further optimize the labeling parameters to clarify the specific contribution of multimerization to radiotracer uptake under conditions with more comparable molar activities.

On the basis of the excellent imaging performance of [^68^Ga]Ga-DOTA-CCK_2_R-dimer, we further evaluated the antitumor potential of its therapeutic counterpart, [^177^Lu]Lu-DOTA-CCK_2_R-dimer. Compared with its monomeric form (e.g., [^177^Lu]Lu-DOTA-CCK-66), the dimer demonstrated superior stability *in vivo*. Urine analysis revealed that more than 96% of the compound remained intact at 1, 4, and 24 h post-injection, indicating strong metabolic stability. In contrast, previous reports showed that only 77.8% of intact [^177^Lu]Lu-DOTA-CCK-66 remained in urine 30 min after injection in mice. This difference may be attributed to the spatial conformational advantages and prolonged circulation time conferred by the dimeric structure, which together enhance metabolic stability. In the AR42J tumor model, [^177^Lu]Lu-DOTA-CCK_2_R-dimer showed sustained radioactivity accumulation in tumors for up to 96 h post-injection, with a tumor uptake of 1.10 ± 0.04 %ID/g, a tumor-to-blood (T/B) ratio of 225.66 ± 40.93, and a tumor-to-muscle (T/M) ratio of 90.85. Notably, relatively higher radioactivity uptake was also observed in non-target organs such as the kidneys and liver, likely due to the larger molecular size and altered pharmacokinetics of the dimer—phenomena that are consistent with other multimeric radiotracers. However, this biodistribution pattern did not compromise the tumor contrast at later time points but rather supports a dynamic balance between efficacy and safety. Taken together, these data show that although variability in the injected dose, molar activity, animal models, and imaging protocols across different studies may affect quantitative comparisons, [^177^Lu]Lu-DOTA-CCK_2_R-dimer exhibits strong structural stability, extended tumor retention, and a favorable biodistribution profile. These characteristics highlight its potential as a promising CCK_2_R-targeted therapeutic agent. Future investigations under standardized experimental conditions, including direct comparisons with representative monomeric tracers (e.g., DOTA-CCK-66 and DOTA-MGS5), as well as dosimetry and toxicity studies, are warranted to further elucidate its clinical translational potential and safety.

One of the key findings of this study is that [^177^Lu]Lu-DOTA-CCK_2_R-dimer exhibits selective toxicity toward poorly differentiated and highly proliferative tumor cells, a feature that has not been previously reported in PRRT. While radiolabeled ligands have been widely applied in the treatment of neuroendocrine tumors and prostate cancer 37, 38, their ability to selectively eliminate these aggressive tumor subpopulations has not been well studied. To address this gap, we employed scRNA-seq to analyze the effects of [^177^Lu]Lu-DOTA-CCK_2_R-dimer on different tumor cell subpopulations. Further differential gene expression analysis revealed that the C4 cell population, which was selectively eliminated by the Combo and Lu treatments, exhibited significant enrichment of key genes associated with chemotherapy resistance (Birc5, Hmgb2, and Rrm1) 39-41, high proliferation (Tuba1b, Stmn1, and Mki67) 42-44, and tumor stemness (Aurka, Lgr5, Hes1, Dnmt1, and Ezh2) 45-47, whereas the C2 cell population did not exhibit these characteristics (**Figure 5D**). The gene set enrichment analysis (GSEA) results revealed that the genes related to the MYC and G2M CHECKPOINT pathways exhibited significant enrichment in the C4 cell population (**Figure 5E**), suggesting that C4 cells are highly proliferative 48-50. In contrast, the C2 cell population did not show significant enrichment of genes in these proliferation-associated pathways, indicating that these cells may be slow cycling or quiescent cells (**Figure 5E**). Pseudotime trajectory analysis was conducted using scVelo with dynamical modeling, revealing that the C3 and C4 cell populations were positioned at the early stage of the tumor differentiation trajectory and represent poorly differentiated tumor cells (**Figure 5F**) and suggesting that they constitute a "stem-like" subpopulation 51. In contrast, the C1 and C2 populations were located at a later stage of the trajectory, indicating a greater degree of differentiation.

To further evaluate the clinical significance of the gene signature of the C4 cell population, we further performed survival analysis using The Cancer Genome Atlas (TCGA) datasets. The results revealed that in pancreatic cancer patients (n = 89), high expression of the top 50 genes in the C4 cell population was significantly associated with shorter disease-free survival (DFS) (HR = 2.0, *p* = 0.002, *p*(HR) = 0.003). Similarly, a significant association was also observed in thyroid cancer patients (n = 255) (HR = 2.1, *p* = 0.013, *p*(HR) = 0.016) (**Figure 5G**). Moreover, a similar trend was observed in patients with paraganglioma (n = 91), but statistical significance was not reached (HR = 2.2, *p* = 0.12, *p*(HR) = 0.13), possibly due to the limited number of cases (**Figure S10**). Previous studies have demonstrated that in treatment-resistant models of lung and pancreatic cancer, cell subpopulations with high expression of HMGB2, BIRC5, and RRM1 exhibit increased DNA damage repair capabilities, leading to resistance to therapies that induce DNA damage such as radiotherapy and certain chemotherapies 18, 40, 41, 52. Consistent with these findings, our data indicate that [^177^Lu]Lu-DOTA-CCK_2_R-dimer can effectively reduce therapy-resistant tumor subpopulations, such as C4, suggesting the potential for improved therapeutic outcomes in aggressive malignancies. Most existing PRRT studies have focused primarily on reducing the overall tumor burden 53, with limited investigations into the role of tumor cell heterogeneity and resistant subpopulations in the treatment response. This study is the first to provide direct evidence that CCK_2_R-targeted radiotherapy can address tumor heterogeneity, highlighting its potential role in precision oncology.

A major finding of this study is the identification of a previously unreported mechanism of radiosensitization in CCK_2_R-targeted radiotherapy. mTOR inhibitors enhance radiosensitivity in various malignancies by inhibiting DNA damage repair, arresting the cell cycle progression, and regulating tumor metabolism, ultimately sensitizing tumor cells to radiation-induced damage 29, 30. Although previous studies, such as that by Grzmil et al., have demonstrated that everolimus-mediated mTOR inhibition can increase CCK_2_R expression and improve the therapeutic response in CCK_2_R-positive xenograft models, the downstream mechanisms by which mTOR inhibition modulates the treatment response remain largely unclear 11. To further elucidate the molecular mechanisms underlying the differential regulation of glutathione metabolism and cytochrome P450-mediated xenobiotic detoxification by [^177^Lu]Lu-DOTA-CCK_2_R-dimer and the mTOR inhibitor (RAD001), we performed a gene screening analysis on the basis of the RNA-seq data. We identified genes that were upregulated in response to Lu treatment and downregulated by mTOR inhibition, but exhibited intermediate or no significant changes in the Combo group, to pinpoint key regulatory factors. Among them, we identified GSTK1 , glutathione peroxidase 1 (GPX1), and glutathione peroxidase 2 (GPX2) in the glutathione metabolism pathway (**Figure 6D**, **Figure S12**). GSTK1 primarily participates in glutathione conjugation-mediated detoxification reactions, while GPX1 and GPX2 play crucial roles in antioxidant defense and radiosensitivity regulation by reducing oxidative damage. Additionally, GSTK1 was also identified in the cytochrome P450-mediated xenobiotic detoxification pathway (**Figure S13**), further emphasizing its key regulatory role in radiotherapy-related detoxification mechanisms. Notably, there was significant enrichment of GSTK1 in both pathways, suggesting that GSTK1 may serve as a key factor in oxidative stress regulation induced by [^177^Lu]Lu-DOTA-CCK_2_R-dimer.

To further assess the clinical significance of GSTK1, we conducted a survival analysis using the TCGA database. The results revealed that in pancreatic cancer patients (n = 89), high GSTK1 expression was significantly associated with shorter overall survival (OS) (HR = 1.6, *p* = 0.027, *p*(HR) = 0.029) (**Figure 6E**). However, GPX1 and GPX2 expression were not significantly correlated with OS (HR = 1.2, *p* = 0.36 for GPX1; HR = 0.99, *p* = 0.97 for GPX2) (**Figure S14**). Additionally, GSTK1 exhibited a moderate-to-strong positive correlation with the top 50 genes of the C4 cell population (R = 0.47, *p* = 5.4e-11; **Figure S15**), suggesting that GSTK1 may be involved in the highly proliferative malignant phenotype of this population. Furthermore, the Western blot (WB) results demonstrated that in cultured AR42J cells, GSTK1 expression was higher in the Lu group than in the Veh group, whereas it was lower in the mTOR group than in the Veh group. Additionally, GSTK1 expression in the Combo group was lower than that in the Lu group (**Figure 6F**), which is consistent with the RNA-seq findings (the uncropped immunoblots with molecular weight standards and loading controls are provided in **Figure S16**). IHC analysis of tumor tissues after 12 days of *in vivo* treatment further confirmed these trends and was in accordance with the WB and RNA-seq results (**Figure 6G**). Additionally, to assess DNA damage, IF staining of γ-H2AX was performed on tumor samples from each treatment group. The strongest γ-H2AX signal was detected in the Combo group, indicating the greatest extent of DNA damage (**Figure 6H**). While prior studies have linked GST family members (e.g., GSTP1 and GSTM1) to radioresistance and oxidative stress regulation 54, GSTK1 has not previously been associated with the radiotherapy response. Our findings reveal a new role of GSTK1 in mitigating radiation-induced oxidative stress and demonstrate that its downregulation via mTOR inhibition enhances radiosensitivity. These results expand our understanding of mTOR signaling in CCK_2_R-targeted radiotherapy and suggest that GSTK1 is a potential target for improving therapeutic efficacy.

Despite the promising radiosensitizing effects of [^177^Lu]Lu-DOTA-CCK_2_R-dimer combined with the mTOR inhibitor RAD001 both *in vitro* and *in vivo*, certain limitations remain. First, this study utilized a mouse xenograft model established with AR42J cells. However, CCK_2_R expression levels and biological functions may vary across different tumor types. Therefore, validation in more clinically representative models, such as patient-derived organoid or patient-derived xenograft (PDX) models, is needed to assess the broader applicability and clinical translatability of this approach. Second, while scRNA-seq revealed the effects of CCK_2_R-targeted radiotherapy on different tumor cell subpopulations, its impact on the tumor microenvironment (TME) remains to be fully elucidated. In particular, the roles of immune cells and fibroblasts in the treatment response and antitumor immunity are not yet well understood. Future studies could incorporate spatial transcriptomics or single-cell ATAC-seq to further explore the interactions between CCK_2_R-targeted radiotherapy and the TME to optimize combination radiotherapy strategies. Additionally, although the mTOR inhibitor effectively radiosensitized the cells in this study, its long-term safety profile needs to be further evaluated. Potential concerns such as metabolic adaptations, immune suppression, and the development of treatment resistance also need to be further investigated in long-term follow-up studies and early phase clinical trials to ensure the safety and sustained efficacy of this therapeutic strategy. One potential limitation of this study lies in the use of equal precursor masses during radiolabeling, which resulted in different molar activities between the monomer and dimer. Nevertheless, previous studies on CCK_2_R-targeted radiotracers have shown that such differences have limited effects on tumor uptake at the doses administered. In future work, we will aim to better match the molar activities to improve the accuracy of quantitative comparisons. Furthermore, the prolonged circulation time of [^68^Ga]Ga-DOTA-CCK_2_R-dimer, while beneficial for tumor targeting, may also lead to increased radiation exposure to the bone marrow, raising potential concerns about hematologic toxicity. This issue warrants further investigation in future dosimetry and long-term safety studies. Finally, the observed high residual uptake in blocking studies, although partially explained by receptor overexpression and tracer affinity, should also be acknowledged as a potential limitation of the current experimental system.

## Conclusion

This study not only fills a research gap by presenting a strategy for CCK_2_R-targeted radiotherapy that targets poorly differentiated and highly proliferative tumor cell populations but also reveals an mTOR inhibitor-based radiosensitization strategy; overall, this study provides experimental evidence and potential clinical applications for optimizing CCK_2_R-targeted radiotherapy. Future research should further explore the impact of CCK_2_R-targeted radiotherapy on the TME and incorporate immunomodulatory strategies to optimize CCK_2_R-targeted radiotheranostic approaches. These findings will facilitate clinical translation and provide a new theoretical foundation and therapeutic strategy for precision CCK_2_R-targeted therapy.

## Supplementary Material

Supplementary materials and methods, figures and tables.
