# Supplementary material for: Dimeric CCK2R radiotheranostic tracers synergize with mTOR inhibition for enhanced tumor therapy
Source: Theranostics. 2025 Aug 16;15(17):9306–25. doi: 10.7150/thno.117021 (PMC12439480; doi:10.7150/thno.117021)
Supplement: Supplementary file 1 — Supplementary materials and methods, figures and tables. [file thnov15p9306s1.pdf]

Supplementary Materials for

Dimeric CCK2R radiotheranostic tracers synergize with mTOR inhibition for enhanced tumor therapy

Authors: Linjie Bian[1-4†], Zheyi Wang[5-6†], Panli Li[1-4†], Simin He[1-4], Jianping Zhang[1-4], Xiaoping Xu[1-4], Xiangwei Wang[1-4*], Shaoli Song[1-4*]

[†]These authors contributed equally to this work.

*Corresponding author(s):

Email: shaoli-song@163.com (S.S.); 20111230008@fudan.edu.cn (X.W.).

This PDF file includes:

Materials and Methods Section S1 to S6

Supplementary Figures S1 to S16

Supplementary Table S1 to S7

Supplementary Methods

Section S1: Chemicals and Instruments

Section S2: *In Vitro* Experiments

Section S3: *In Vivo* Experiments

Section S4: Single-Cell RNA Sequencing and Immunohistochemical (IHC) Analysis of Mouse Tumor Tissue

Section S5: Reactive oxygen species (ROS) detection, RNA Sequencing Analysis, and Western Blotting (WB)

Section S6: Toxicity Studies

Section S1: Chemicals and Instruments

Chemicals

NH_4OAc, ACN, CH_3COH, and HCl (37%) (China National Medicines Corporation Ltd.), NaOH (Bide Medical Company, China), sodium chloride injection (China Otsuka Pharmaceutical Co., Ltd.), 2-ethyl hexanol (Shanghai Macklin Biochemical Technology Co., Ltd., China), CCK_2R antibody (4A5, Santa Cruz Biotechnology, USA), fetal bovine serum (FBS, Moregate Biotech, Australia), phosphate-buffered saline (PBS, Gibco, USA), bovine serum albumin (BSA, Sigma, USA), protease inhibitors (P8340, Sigma Life Sciences, USA) , reactive oxygen species(ROS) assay kit (S0033S, Beyotime Biotechnology), and anti-GSTK1 antibody (No. A5226, ABclonal).

Instruments

Inveon PET-CT scanner (Siemens AG, Germany), γ-counter (Shanghai Hesuo Rihuan Photoelectric Instrument Ltd., China), and cell culture incubator (Thermo Fisher Scientific, USA).

S2: *In Vitro* Experiments

Cell Culture

The rat pancreatic exocrine AR42J cell line (CCK_2R expressing)[1-5] was obtained from Shanghai Institute of Biochemistry and Cell Biology, Chinese Academy of

Sciences. Cells were cultured in F-12K medium supplemented with 20% (v/v) fetal bovine serum (FBS) and 1% (v/v) penicillin-streptomycin, maintained at 37°C in a humidified 5% CO_2 atmosphere. Cell line authentication was performed using short tandem repeat (STR) profiling, with mycoplasma contamination testing conducted triweekly.

References

1. Di Santo G, *et al.* Cholecystokinin-2 receptor targeting by [(68)Ga]Ga-DOTA-MGS5 PET/CT in a patient with extensive disease small cell lung cancer. *Eur J Nucl Med Mol Imaging* **51**, 2848-2849 (2024).
2. Zavvar TS, *et al.* Radiopharmaceutical formulation and preliminary clinical dosimetry of [(177)Lu]Lu-DOTA-MGS5 for application in peptide receptor radionuclide therapy. *Eur J Nucl Med Mol Imaging* **52**, 1321-1331 (2025).
3. Günther T, *et al.* Preclinical Evaluation of Minigastrin Analogs and Proof-of-Concept [(68)Ga]Ga-DOTA-CCK-66 PET/CT in 2 Patients with Medullary Thyroid Cancer. *J Nucl Med* **65**, 33-39 (2024).
4. von Guggenberg E, *et al.* Safety, Biodistribution, and Radiation Dosimetry of the (68)Ga-Labeled Minigastrin Analog DOTA-MGS5 in Patients with Advanced Medullary Thyroid Cancer and Other Neuroendocrine Tumors. *J Nucl Med* **66**, 257-263 (2025).
5. Rottenburger C, *et al.* Cholecystokinin 2 Receptor Agonist (177)Lu-PP-F11N for Radionuclide Therapy of Medullary Thyroid Carcinoma: Results of the Lumed Phase 0a Study. *J Nucl Med* **61**, 520-526 (2020).

Cell Uptake and Inhibition Experiments

Cell uptake studies were performed using the AR42J cell line. AR42J cells (5×10^4 cells/well) were seeded into 24-well plates one day prior to the experiment. After removing the medium, the cells were washed twice with PBS. Subsequently, 500 μL of fresh medium containing 37 kBq of [^{68}Ga]Ga-labeled radiotracers (e.g., [^{68}Ga]Ga-DOTA-CCK_2R-dimer and [^{68}Ga]Ga-DOTA-CCK-66) and 500 μL of fresh medium containing 37 kBq of [^{177}Lu]Lu-labeled radiotracer ([^{177}Lu]Lu-DOTA-CCK_2R-dimer) were added separately and incubated with the cells under sterile conditions at 37°C for 30, 60, and 120 minutes. After incubation, the medium was removed, and the cells were washed twice with 1 mL of cold PBS, followed by lysis with 500 μL of 1 M NaOH. The NaOH solution was collected, and the radioactivity was measured using a γ-counter.

To further evaluate the targeting specificity of the radiotracers, their uptake in AR42J cells was measured at the 1-h time point in the presence of a 400-fold excess

of the lead compound.

S3: *In vivo* Experiments

Mouse Models

All animal studies were approved by the Ethics Committee of Fudan University Shanghai Cancer Center (No. FUSCC-IACUC-S2023-0003). Five-week-old female BALB/c nude mice were purchased from Shanghai SLAC Laboratory Animal Company. To establish cell-derived xenograft (CDX) models, 1 × 10^6 AR42J cells (100 μL) were subcutaneously inoculated into the right shoulder of each mouse. Imaging and biodistribution studies were conducted once the tumor diameter reached 0.5 - 1 cm.

Pharmacokinetics in Normal Mice

For the pharmacokinetics study, six male KM mice (4 - 6 weeks old, weighing 18 - 20 g) were administered either [^{68}Ga]Ga-DOTA-CCK$_2$R-dimer or [^{68}Ga]Ga-DOTA-CCK-66 (3.7 MBq per mouse, n = 5/group) via tail vein injection. Blood samples were collected from the tail vein using pre-weighed capillary tubes at 1, 3, 5, 10, 15, 30, 60, 90, 120, and 150 minutes after radiotracer injection. Radioactivity levels were measured using a gamma counter, with decay correction applied based on the injection time. The percentage of injected dose per gram of tissue (%ID/g) was calculated for each time point.

Section S4: Single-Cell RNA Sequencing and Immunohistochemical (IHC) Analysis of Mouse Tumor Tissue

Single-Cell RNA Sequencing

Cell preparation

To isolate single cells from AR42J tumors, tumor tissues were collected from mice in different treatment groups. The tumor tissues were dissected and washed multiple times with pre-cooled PBS to remove necrotic content. Subsequently, the tissues were minced into small pieces and incubated with 0.5 mg/mL digestive enzyme 1 (provided by 10K Genomics) at 37 °C in a water bath for 30 minutes. After

incubation, the mixture was centrifuged, and the supernatant was collected. The remaining pellet was subjected to a second round of digestion with 0.5 mg/mL digestive enzyme 1 for another 30 minutes. After a total digestion time of 60 minutes, the supernatant and pellet were combined and filtered through a 70 μM cell strainer (BD Falcon, 352350). The mixture was then centrifuged at 500 g for 5 minutes. The resulting pellet was further digested with 1 mL of trypsin for 15 minutes. The supernatant was collected and filtered through a 35 μM cell strainer (BD Falcon, 352235). After another centrifugation at 500 g for 5 minutes, the cell pellet was resuspended in PBS containing 0.01% BSA.

Single-Cell RNA Sequencing (scRNA-seq)

The cell suspension was loaded into Chromium microfluidic chips with 3' chemistry and barcoded with a 10× Chromium Controller (10X Genomics). RNA from the barcoded cells was subsequently reverse-transcribed and sequencing libraries constructed with reagents from a Chromium Single Cell 3' v2 reagent kit (10X Genomics) according to the manufacturer's instructions. Sequencing was performed with Illumina (HiSeq 2000) according to the manufacturer's instructions (Illumina).

Data analysis

Qualitycontrol

We use fastp to perform basic statistics on the quality of the raw reads. Generally, cellranger count support FASTQ files from raw base call (BCL) files generated by Illumina sequencers as input file. 10x Genomics® not recommend additional processing of the sequence. If cleanreads is indispensable: Then, raw read sequences produced by the Illumina pipeline in FASTQ format were pre-processed through Trimmomatic software which can be summarized as below:

(1) Remove low-quality reads: Scan the read with a a4-base wide sliding window, cutting when the average quality per base drops below 10 (SLIDING WINDOW: 4 : 10)

(2) Remove trailing low quality or N bases (below quality 3) (TRAILING: 3)

(3) Remove adapters: There are two modes to remove the adapter sequence: a. alignment with the adapter sequence, the number of matching bases was more

significant than 7 and mismatch = 2; b. when read1 and read2 overlapping base scoring greater than 30, removed non-overlapping portions (ILLUMINACLIP: adapter.fa: 2 : 30 : 7)

(4) Drop reads below the 26 bases long (5) Discard those reads that can not form paired. The remaining reads that passed all the filtering steps were counted as clean reads, and all subsequent analyses were based on this. At last, we use fastp to perform basic statisticson on the quality of the clean reads.

Generation and Analysis of Single-Cell Transcriptomes

Raw reads were demultiplexed and mapped to the reference genome by 10X Genomics Cell Ranger pipeline (https://support.10xgenomics.com/single-cell-gene expression/software/pipelines/latest/what-is-cell-ranger) using default parameters. All downstream single-cell analyses were performed using Cell Ranger and Seurat. In brief, for each gene and each cell barcode (filtered by CellRanger), unique molecule identifiers were counted to construct digital expression matrices.

In detail: cellranger count takes FASTQ files performs alignment, filtering, barcode counting, and UMI counting. It uses the Chromium cellular barcodes to generate feature barcode matrices, determine clusters, and perform gene expression analysis. The count pipeline can take input from multiple sequencing runs on the same GEM well. Add-–nosecondary option to skip secondary analysis of the feature-barcode matrix (dimensionality reduction, clustering and visualization).

When doing large studies involving multiple GEM wells, run cellranger count on FASTQdata from each of the GEM wells individually, and then pool the results using cellranger aggr:

cellranger aggr aggregates outputs from multiple runs of cellranger count, normalizing those runs to the same sequencing depth and then recomputing the feature-barcode matrices and analysis on the combined data. The aggr pipeline can be used to combine data from multiple samples into an experiment-wide feature-barcode matrix and analysis.

Secondary Analysis of Gene Expression

Seurat

The Seurat package was used to normalise data, dimensionality reduction, clustering, differential expression. And batch effects was corrected using the R package HARMONY (Version 0.1.1) before clustering analysis in Seurat (Version 4.3.0.1). All cells expressing <200 or >8,000 genes were removed, as well as cells that contained <500 or >50000 unique molecular identifiers (UMIs/counts) and >15% mitochondrial counts. Samples filtered the double cells through DoubletFinder (Version 2.0.3) in github ('chris-mcginnis-ucsf/DoubletFinder'). Then, the elbow plot was generated using the ElbowPlot function in Seurat, based on which the number of significant principal components (PCs) was determined to be 10. The resolution parameter of the FindClusters function varied across different cell types, set to 0.15 for all cells to obtain 7 tumor subclusters. Mixed immune cells from the nude mice's microenvironment were excluded based on conventional markers.

To explore the function of different clusters, we calculated the glutathione transfer score and MTOR-dependent signature using the AddModuleScore function in Seurat for individual cells across groups, respectively.

Statistical analyses

All statistical analyses were performed using R version 4.3.1 (http://www.r-project.org). A nonparametric two-sided Wilcoxon rank-sum test was used to compare two populations unless the data followed normal distributions, in which case a two-sided t-test was applied. For all tests, a p-value <0.05 was considered statistically significant.

Correlation analyses were performed using Pearson correlation with associated two-sided p-values, and visualized with linear graphs through RStudio. The co-occurrence relationships between two cell types and two genes were analyzed using Spearman's correlation coefficients.

Enrichment analysis of marker genes

Gene Ontology (GO) enrichment analysis of marker genes was implemented by the clusterProfiler R package, in which gene length bias was corrected. GO terms with corrected Pvalue less than 0.05 were considered significantly enriched by marker gene.

KEGG is a database resource for understanding high-level functions and utilities of the biological system, such as the cell, the organism and the ecosystem, from molecular-level information, especially large-scale molecular datasets generated by genome sequencing and other high-through put experimental technologies (http://www.genome.jp/kegg/). We use dclusterProfiler R package to test the statistical enrichment of markergenes in KEGG pathways.

Reactome pathway-based analysis of marker genes was implemented by the ReactomePA R package. REACTOME is an open-source, open access, manually curated and peer-reviewed pathway database(https://reactome.org/) .

RNA Velocity Analysis

First, the velocity software was used to generate a loom file from the sorted bam file and the genome annotation file "GRCmm10.gtf". After that, the loom files of each sample were merged into a single loom file. The R package sceasy was used to convert the cell Seurat files of each subpopulation into h5ad files, and we used the scanpy package of Python to generate the AnnData object, based on the scVelo algorithm, and the Python package scVelo and Cellrank for RNA velocity analysis to calculate the RNA velocity and plot the visualization.

TCGA analysis based on GEPIA2

GEPIA2 is for analyzing the RNA sequencing expression data of pancreatic and thyroid cancer cohorts from the TCGA and the GTEx projects, using a standard processing pipeline(http://gepia2.cancer-pku.cn/). And we use the Survival Analysis and Correlation Analysis parts for study the C4 clusters features and GSTK1.

Immunohistochemistry (IHC)

On day 12 post-treatment, tumor tissues were collected for IHC and immunofluorescence analysis to assess GSTK1 and γ-H2AX expression.

IHC Analysis of GSTK1 Expression

Tumor tissue sections (4 μm) were deparaffinized, rehydrated, and subjected to antigen retrieval by heating in citrate buffer (pH 6.0) at 95°C for 20 minutes. Endogenous peroxidase activity was blocked with 3% hydrogen peroxide, followed by blocking with 5% BSA for 30 minutes. The sections were incubated overnight

with a recombinant anti-GSTK1 primary antibody (1:50) at 4°C. After PBS washing, an HRP-conjugated secondary antibody was applied for 30 minutes at room temperature. Detection was performed using a DAB substrate, followed by hematoxylin counterstaining. The sections were then dehydrated, mounted, and examined under a light microscope.

γ-H2AX Immunofluorescence Staining

After antigen retrieval, sections were blocked with 5% BSA for 10-20 minutes. The primary anti-γ-H2AX antibody (1:50) was applied and incubated at room temperature for 1 hour or overnight at 4°C. After PBS washing, Alexa Fluor 488-conjugated secondary antibody (1:500) was added and incubated at room temperature for 1 hour. The sections were then washed, counterstained with DAPI for 5 minutes, mounted, and observed under a fluorescence microscope to visualize γ-H2AX (green) and DAPI (blue).

Section S5: Reactive oxygen species (ROS) detection, RNA Sequencing Analysis, and Western Blot (WB)

Reactive oxygen species (ROS) detection

AR42J cells were divided into four groups and incubated in serum-free medium under the following conditions: (1) [^{177}Lu]Lu-DOTA-CCK$_2$R-dimer (3.7 MBq/mL), (2) RAD001 (10 μM), (3) a combination of [^{177}Lu]Lu-DOTA-CCK$_2$R-dimer (3.7 MBq/mL) and RAD001 (10 μM), and (4) a control group without any treatment. Cells were incubated at 37°C in a humidified atmosphere of 5% CO_2 for 24 hours. After incubation, reactive oxygen species (ROS) levels were assessed using the DCFH-DA probe and flow cytometry. Cells were incubated with DCFH-DA, which is oxidized to fluorescent DCF upon interaction with ROS. The fluorescence intensity was then measured by flow cytometry to quantify intracellular ROS levels.

RNA Sequencing Analysis

RNA Extraction, Library Preparation, and Transcriptome Sequencing

AR42J cells were divided into four groups and incubated in serum-free medium under the following conditions: (1) [^{177}Lu]Lu-DOTA-CCK$_2$R-dimer (3.7 MBq/mL),

(2) RAD001 (10 μM), (3) a combination of [^{177}Lu]Lu-DOTA-CCK$_2$R-dimer (3.7 MBq/mL) and RAD001 (10 μM), and (4) a control group without any treatment. Cells were incubated at 37°C in a humidified atmosphere of 5% CO_2 for 24 hours, after which total RNA was extracted for transcriptome analysis. RNA integrity was evaluated using the Bioanalyzer 2100 system (Agilent Technologies, CA, USA). Messenger RNA (mRNA) was isolated from total RNA using poly-T oligo-attached magnetic beads. The mRNA was fragmented and reverse-transcribed into first-strand cDNA using random hexamer primers, followed by second-strand synthesis incorporating dUTP instead of dTTP for directional library preparation. Libraries were constructed using end repair, A-tailing, adapter ligation, size selection, USER enzyme digestion, amplification, and purification. Quantification was performed with Qubit and real-time PCR, and the size distribution of libraries was verified using a bioanalyzer. The sequencing data were processed using featureCounts v1.5.0-p3 to quantify reads mapped to individual genes. Gene expression levels were estimated by calculating FPKM (Fragments Per Kilobase of transcript per Million mapped reads), which considers both sequencing depth and gene length, ensuring reliable expression quantification.

Differentially Expressed Gene (DEG) Analysis

Differentially expressed genes (DEGs) were identified using DESeq2 (version 1.40.2). For the analysis of combo treatment effects on the glutathione metabolic pathway, candidate genes were selected based on the following criteria: in the Lu vs Veh comparison, genes with log2FC > 0.5 and P-value < 0.05 were classified as significantly upregulated; in the mTOR vs Veh comparison, genes with log2FC < -0.5 and P-value < 0.05 were categorized as downregulated; while genes with |log2FC| < 0.5 in the Combo vs Veh comparison were independently analyzed using ggVennDiagram to identify potential interaction effects. The analysis aimed to uncover key molecular changes underlying the therapeutic effects of [^{177}Lu]Lu-DOTA-CCK$_2$R-dimer and mTOR inhibition.

Functional Enrichment Analysis

To explore the biological significance of DEGs, we performed Gene Ontology

(GO) enrichment analysis, Kyoto Encyclopedia of Genes and Genomes (KEGG) pathway analysis, and Reactome pathway analysis. GO enrichment analysis was conducted using clusterProfiler (v4.0.5). KEGG pathway enrichment was analyzed using clusterProfiler, focusing on pathways relevant to radiotherapy-related detoxification, including glutathione metabolism and cytochrome P450-mediated metabolism of xenobiotics. Reactome pathway analysis was performed using ReactomePA (v1.34.0) to identify key metabolic and signaling pathways contributing to radiosensitivity regulation.

Western Blot Analysis

AR42J cells were divided into four groups and treated with the following reagents in serum-free medium: (1) [^{177}Lu]Lu-DOTA-CCK$_2$R-dimer (3.7 MBq/mL), (2) RAD001 (10 μM), (3) a combination of [^{177}Lu]Lu-DOTA-CCK$_2$R-dimer (3.7 MBq/mL) and RAD001 (10 μM), and (4) a control group with no treatment. After incubation at 37°C with 5% CO_2 for 24 hours, proteins were extracted using a lysis buffer containing 150 mM NaCl, 50 mM Tris-HCl (pH 8.0), 1 mM EDTA, and 1% protease and phosphatase inhibitors. Total protein (20 μg per sample) was separated on SDS-PAGE gels and transferred to PVDF membranes (Millipore). Membranes were blocked with 5% skimmed milk in TBST for 1 hour at room temperature and then incubated overnight at 4°C with a mouse anti-GSTK1 antibody (1:500 dilution, ABclonal, Cat. No. A5226). After three washes with TBST, membranes were incubated with an HRP-conjugated secondary antibody for 1 hour at room temperature. Protein bands were visualized using an enhanced chemiluminescence detection system (CLINX, ChemiScope 6200).

Section S6: Toxicity Studies

Blood samples were collected retro-orbitally using a glass capillary tube when mice reached one of the predefined endpoints. The samples were then centrifuged at 6,000 rpm for 10 minutes (Centrifuge 5417R, Eppendorf SE, Enfield, CT, USA), and the supernatant was transferred into a preventive care profile plus rotor for analysis of RBC, WBC, PLT, ALT, AST, ALP, BUN, and CREA. Sample analysis was performed

following the standard operating procedures of the VetScan VS2 system (Abaxis, Union City, CA, USA).

Once the animals reached one of the predefined endpoints, organs (heart, liver, spleen, kidneys) from three randomly selected mice per group were collected and subjected to hematoxylin and eosin (H&E) staining. The samples were processed and analyzed using a Leica Bond RX processor (Leica Biosystems, Deer Park, TX, USA).

Supplementary Figures

Supplementary Figure S1. Chemical structure and mass spectrum of DOTA-CCK-66 and DOTA-CCK$_2$R-dimer.

Supplementary Figure S2. HPLC Characterization of [^{68}Ga]Ga-DOTA-CCK$_2$R-dimer, [^{177}Lu]Lu-DOTA-CCK$_2$R-dimer, and [^{68}Ga]Ga-DOTA-CCK-66.

Supplementary Figure S3. Partition Coefficients of [^{68}Ga]Ga-DOTA-CCK$_2$R-dimer and [^{68}Ga]Ga-DOTA-CCK-66.

Supplementary Figure S4. Stability of [^{68}Ga]Ga-DOTA-CCK$_2$R-dimer and [^{68}Ga]Ga-DOTA-CCK-66.

Supplementary Figure S5. Pharmacokinetic Profiles of [^{68}Ga]Ga-DOTA-CCK$_2$R-dimer and [^{68}Ga]Ga-DOTA-CCK-66.

Supplementary Figure S6. Cellular Uptake and Blocking Studies of [^{68}Ga]Ga-DOTA-CCK$_2$R-dimer and [^{68}Ga]Ga-DOTA-CCK-66.

Supplementary Figure S7. Cellular Uptake and Blocking Studies of [^{177}Lu]Lu-DOTA-CCK2R-dimer.

Supplementary Figure S8. *In vitro* and *in vivo* stability of [^{177}Lu]Lu-DOTA-CCK$_2$R-dimer.

Supplementary Figure S9. Uniform distribution of cell populations across different treatment groups.

Supplementary Figure S10. Relationship between C4 cell cluster gene characteristics and progression-free survival (DFS) in paraganglioma patients.

Supplementary Figure S11. Single-cell characterization of RAD001-sensitive mTOR-dependent gene signatures in tumor subclusters.

Supplementary Figure S12. Bulk RNA-seq analysis of glutathione S-transferase family members (GSTK1, GPX1, and GPX2) expression across therapeutic interventions (Veh, Lu, mTOR, and Combo).

Supplementary Figure S13. Venn diagram of differentially expressed genes (DEGs) in cytochrome P450 (CYP)-mediated xenobiotic detoxification pathway across treatment comparisons.

Supplementary Figure S14. Kaplan-Meier analysis of (a) GPX1 and (b) GPX2 in pancreatic cancer patients from the TCGA PAAD cohort (n = 89).

Supplementary Figure S15. Scatter plot showing correlation between GSTK1 expression and top 50 C4-associated genes.

Supplementary Figure S16. Uncropped western blot images showing GSTK1 protein expression in AR42J cells under four treatment conditions.

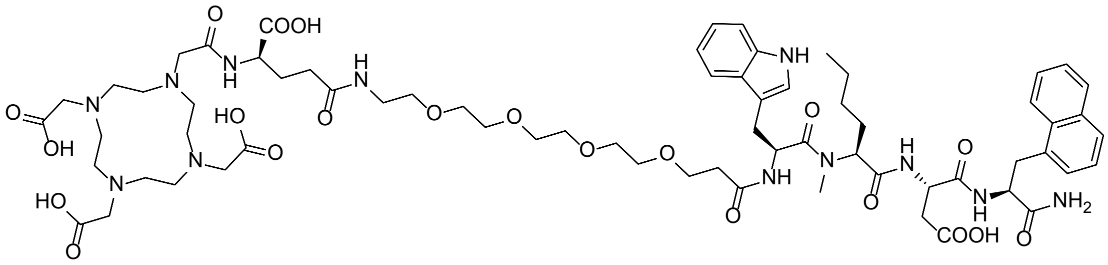

Supplementary Figure S1a. Chemical structure of DOTA-CCK-66.

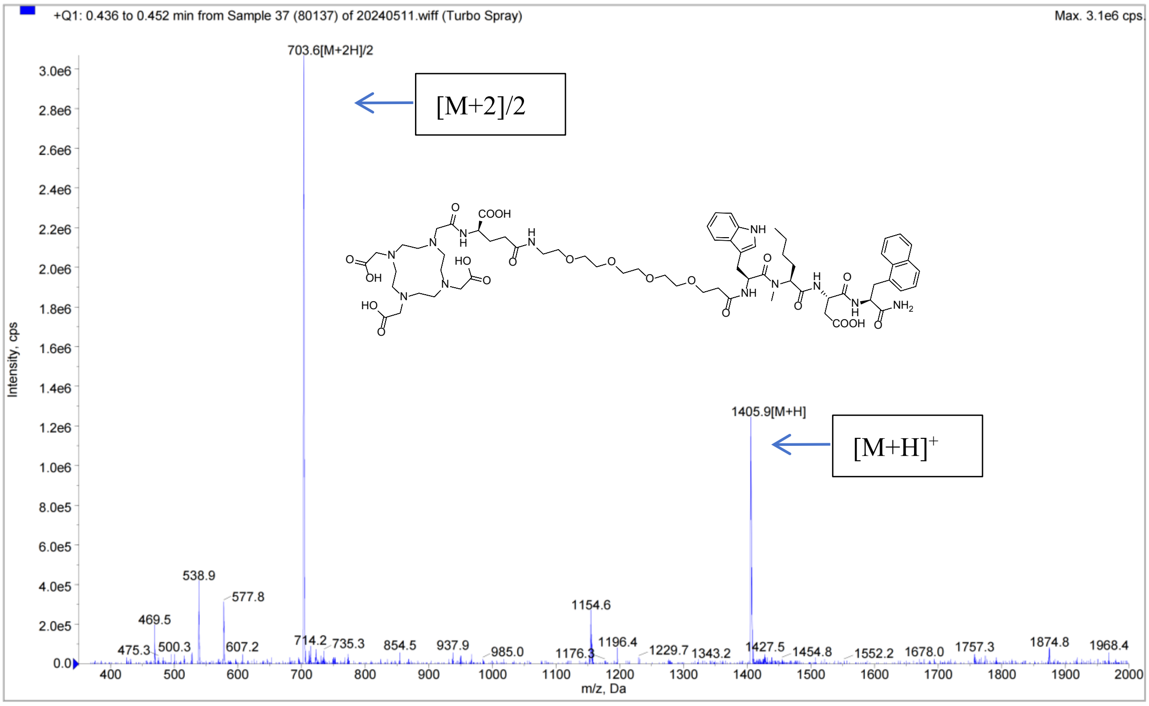

Figure S1b. MS spectrum of compound DOTA-CCK-66. MS-ESI(+) calculated for C67H96N12O21: 1404.6813, [M+H]+ found: 1405.9, [M+2]/2 found: 703.6.

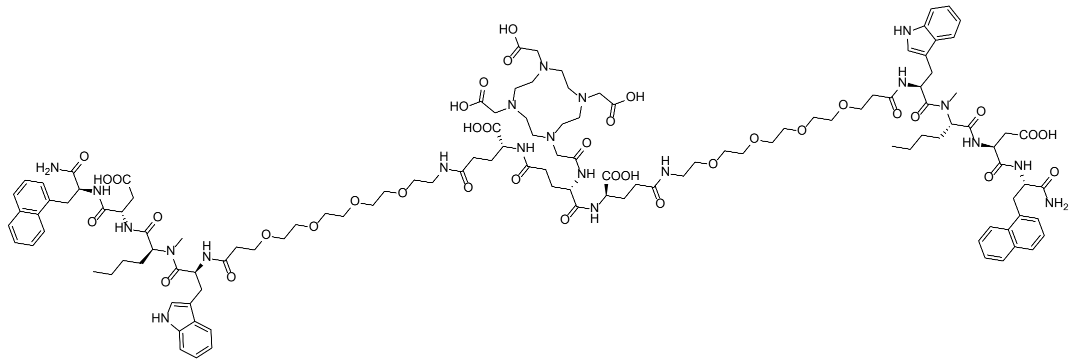

Figure S1c. Chemical structure of DOTA-CCK$_2$R-dimer.

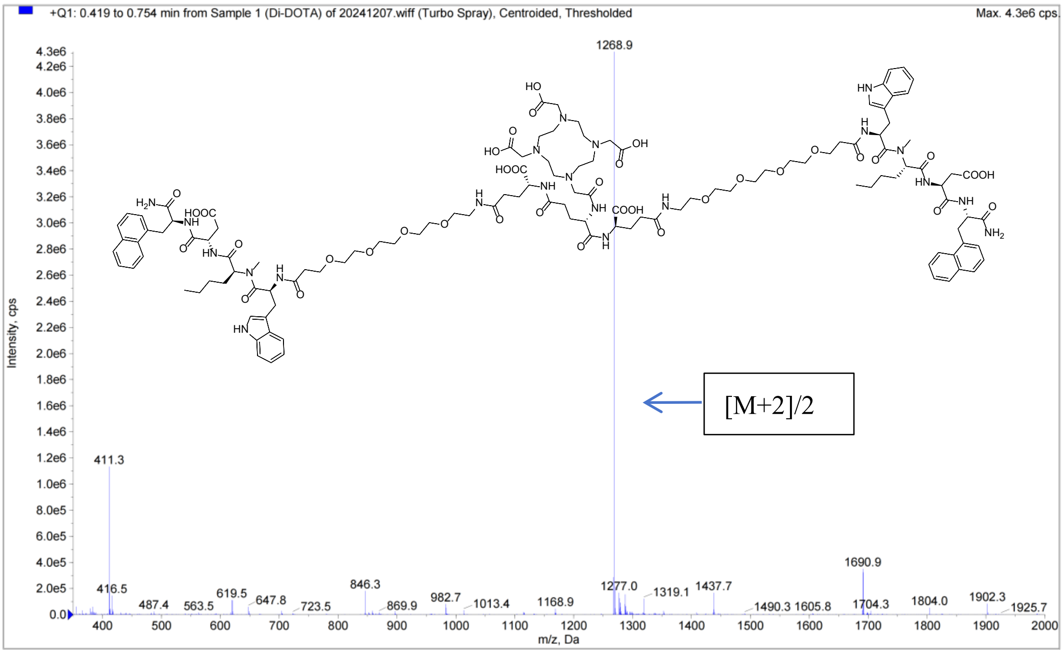

Figure S1d. MS spectrum of compound DOTA-CCK$_2$R-dimer. MS-ESI(+) calculated for $C_{123}H_{171}N_{21}O_{37}$: 2534.2145, [M+2]/2 found: 1268.9.

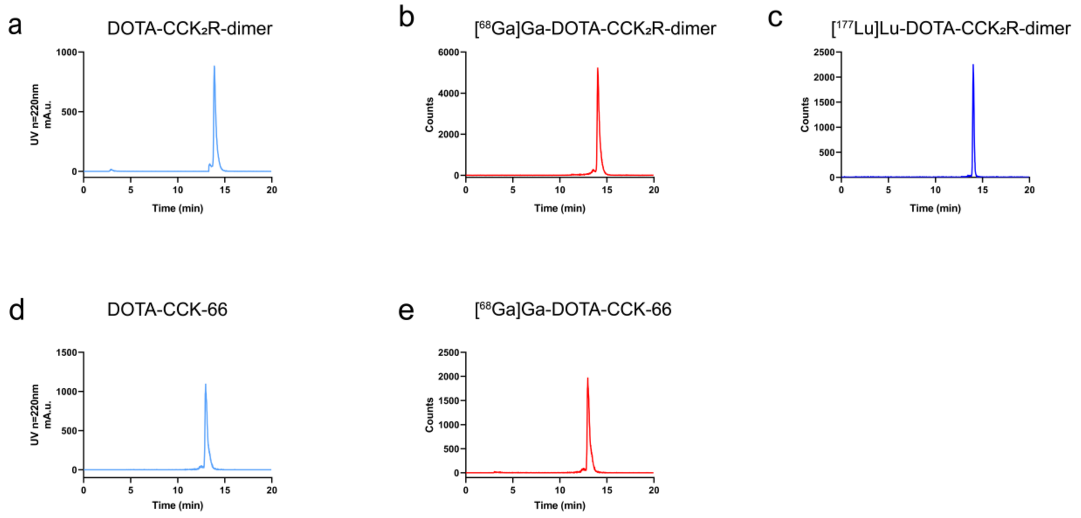

Supplementary Figure S2. HPLC Characterization of [^{68}Ga]Ga-DOTA-CCK$_2$R-dimer, [^{177}Lu]Lu-DOTA-CCK$_2$R-dimer, and [^{68}Ga]Ga-DOTA-CCK-66.

(a) Representative HPLC chromatogram of the precursor DOTA-CCK$_2$R-dimer.

(b) Representative radio-HPLC chromatogram of [^{68}Ga]Ga-DOTA-CCK$_2$R-dimer.

(c) Representative radio-HPLC chromatogram of [^{177}Lu]Lu-DOTA-CCK$_2$R-dimer.

(d) Representative HPLC chromatogram of the precursor DOTA-CCK-66.

(e) Representative radio-HPLC chromatogram of [^{68}Ga]Ga-DOTA-CCK-66.

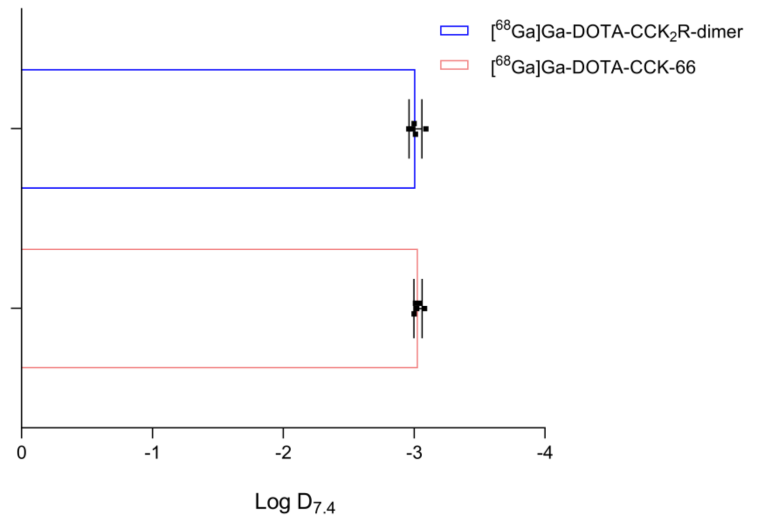

Supplementary Figure S3. Partition Coefficients of [^{68}Ga]Ga-DOTA-CCK$_2$R-dimer and [^{68}Ga]Ga-DOTA-CCK-66. The Log $D_{octanol/PBS}$ values for [^{68}Ga]Ga-DOTA-CCK$_2$R-dimer and [^{68}Ga]Ga-DOTA-CCK-66, representing their hydrophilicity in a physiological environment.

Data are presented as mean ± SD (n = 5).

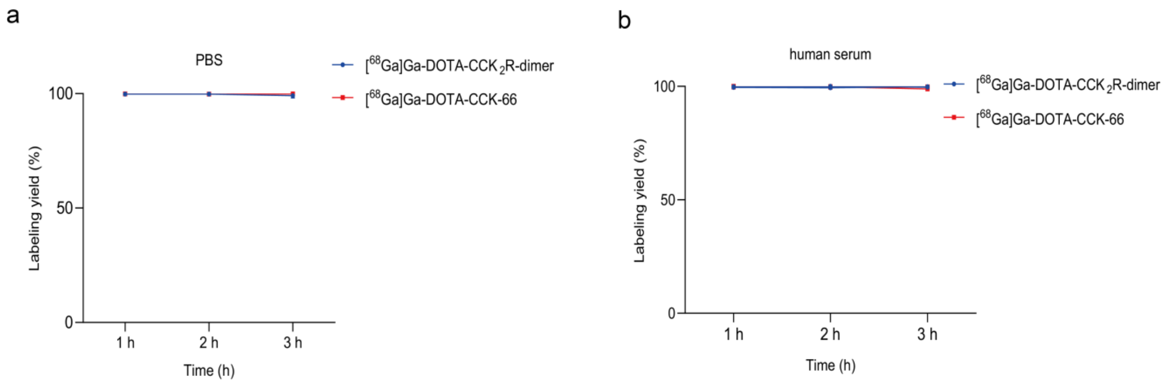

Supplementary Figure S4. Stability of [^{68}Ga]Ga-DOTA-CCK$_2$R-dimer and [^{68}Ga]Ga-DOTA-CCK-66.

(a) Stability of [^{68}Ga]Ga-DOTA-CCK$_2$R-dimer and [^{68}Ga]Ga-DOTA-CCK-66 in PBS at 1, 2, and 3 h, analyzed using radio-HPLC.

(b) Stability of [^{68}Ga]Ga-DOTA-CCK$_2$R-dimer and [^{68}Ga]Ga-DOTA-CCK-66 in human serum at 1, 2, and 3 h, analyzed using radio-HPLC.

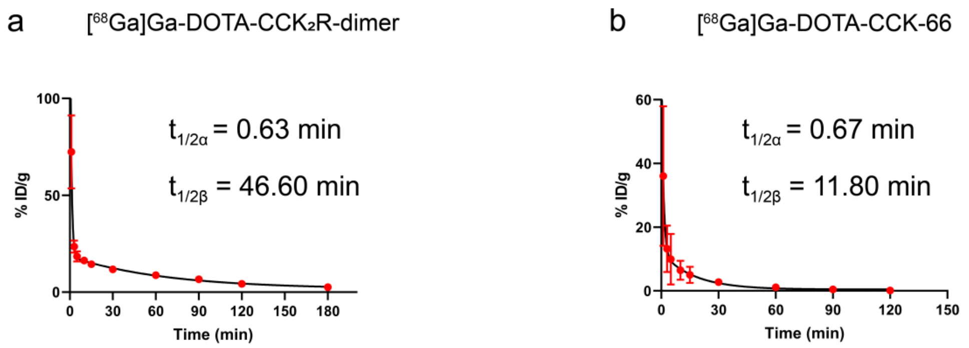

Supplementary Figure S5. Pharmacokinetic Profiles of [^{68}Ga]Ga-DOTA-CCK$_2$R-dimer and [^{68}Ga]Ga-DOTA-CCK-66.

(a) Blood clearance profile of [^{68}Ga]Ga-DOTA-CCK$_2$R-dimer (n = 5).

(b) Blood clearance profile of [^{68}Ga]Ga-DOTA-CCK-66 (n = 5).

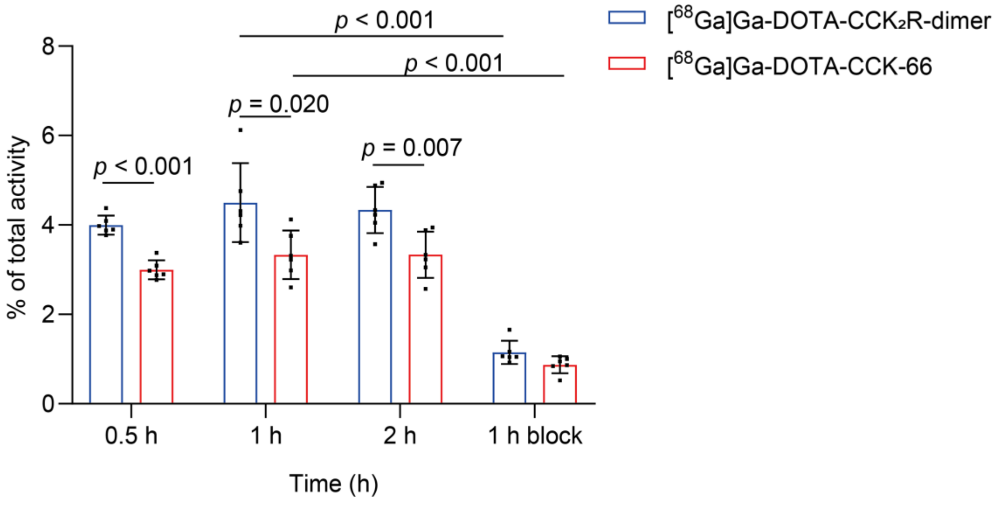

Supplementary Figure S6. Cellular Uptake and Blocking Studies of [^{68}Ga]Ga-DOTA-CCK$_2$R-dimer and [^{68}Ga]Ga-DOTA-CCK-66. The bar graph illustrates the cellular uptake of [^{68}Ga]Ga-DOTA-CCK$_2$R-dimer and [^{68}Ga]Ga-DOTA-CCK-66 in AR42J cells at 0.5, 1, and 2 h. At all time points, [^{68}Ga]Ga-DOTA-CCK$_2$R-dimer exhibited significantly higher uptake than [^{68}Ga]Ga-DOTA-CCK-66 ($p < 0.05$). Additionally, the blocking study conducted at 1 h demonstrated that co-incubation with excess unlabeled ligand significantly reduced the uptake of both radiotracers, confirming their receptor-specific binding ($p < 0.05$). Data are presented as mean ± SD (n = 5).

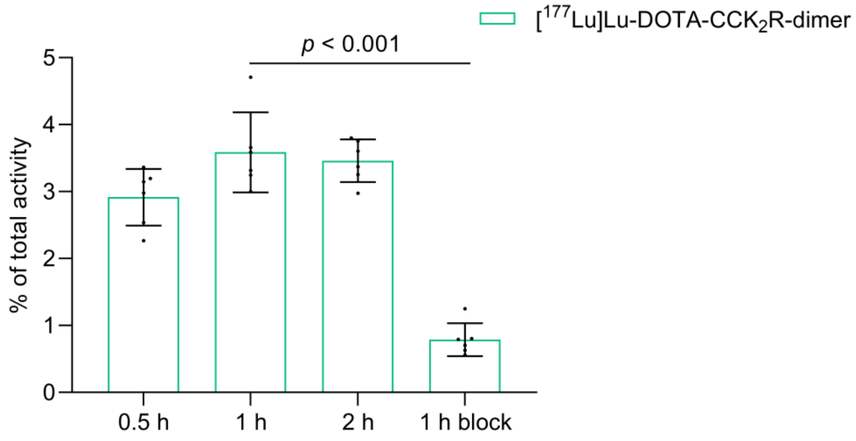

Supplementary Figure S7. Cellular Uptake and Blocking Studies of [^{177}Lu]Lu-DOTA-CCK$_2$R-dimer. The bar graph illustrates the cellular uptake of [^{177}Lu]Lu-DOTA-CCK2R-dimer in AR42J cells at 0.5, 1, and 2 h. The blocking study conducted at 1 h demonstrated that co-incubation with excess unlabeled ligand significantly reduced tracer uptake, confirming receptor-specific binding ($p < 0.05$). Data are presented as mean ± SD (n = 5).

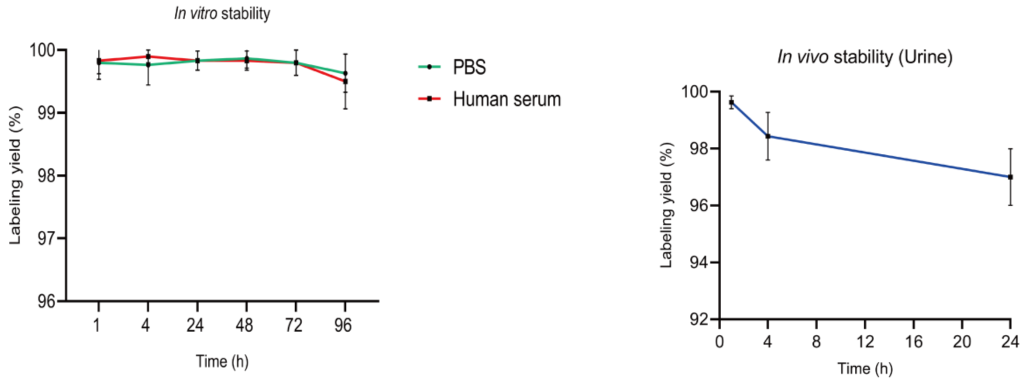

Supplementary Figure S8. ***In vitro*** **and** ***in vivo*** **stability of [^{177}Lu]Lu-DOTA-CCK$_2$R-dimer.** The left panel shows the *in vitro* stability of [^{177}Lu]Lu-DOTA-CCK$_2$R-dimer in phosphate-buffered saline (PBS) and human serum at 1, 4, 24, 48, 72, and 96 hours post-incubation, with radiochemical purity remaining above 99% throughout. The right panel presents the *in vivo* stability based on urine samples collected at 1, 4, and 24 hours post-injection, showing maintained radiochemical purity above 96%, indicating excellent tracer integrity *in vivo*.

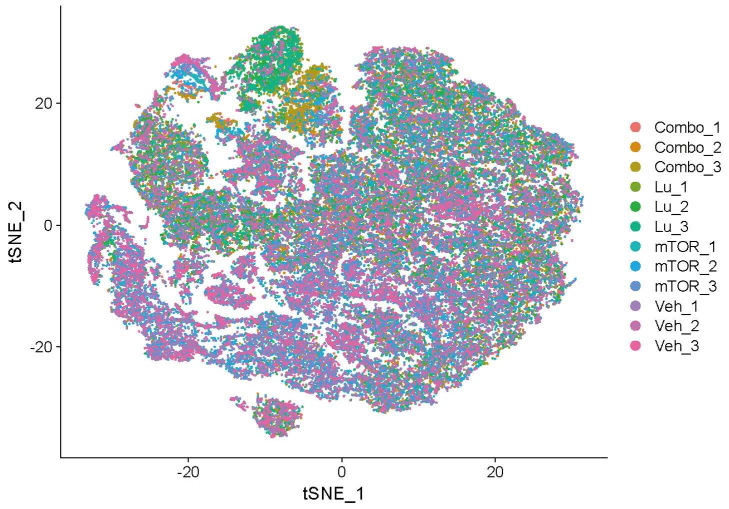

Supplementary Figure S9. Uniform distribution of cell populations across different treatment groups. The UMAP (Uniform Manifold Approximation and Projection) plot illustrates the distribution of cells from different treatment groups. The clustering pattern indicates that cell populations are evenly distributed across groups, with no significant batch effects observed.

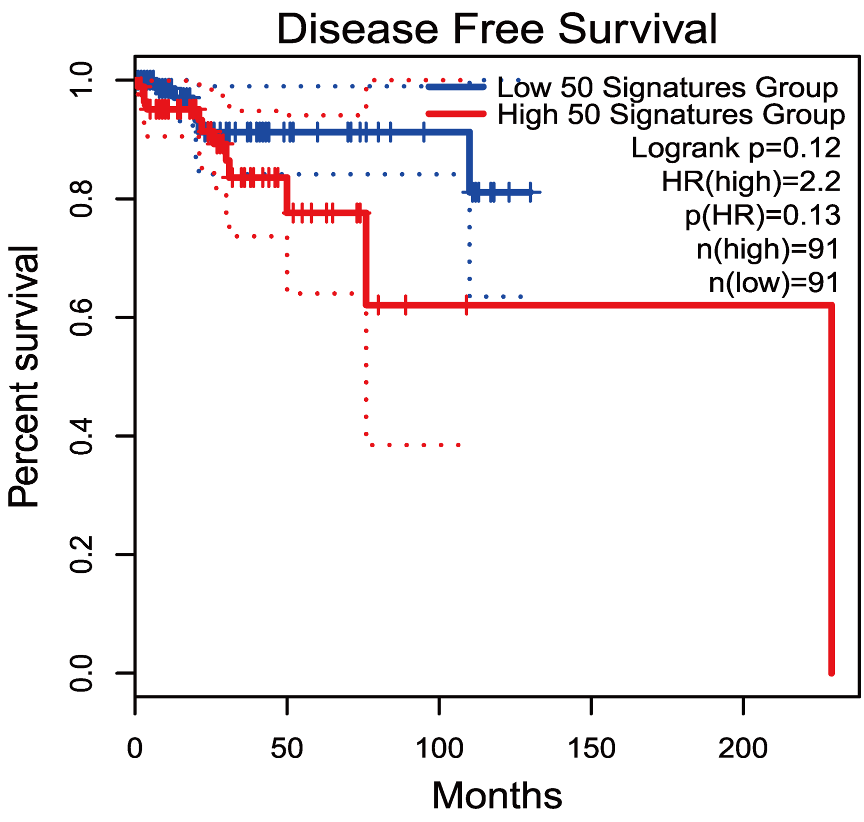

Supplementary Figure S10. Relationship between C4 cell cluster gene characteristics and progression-free survival (DFS) in paraganglioma patients.

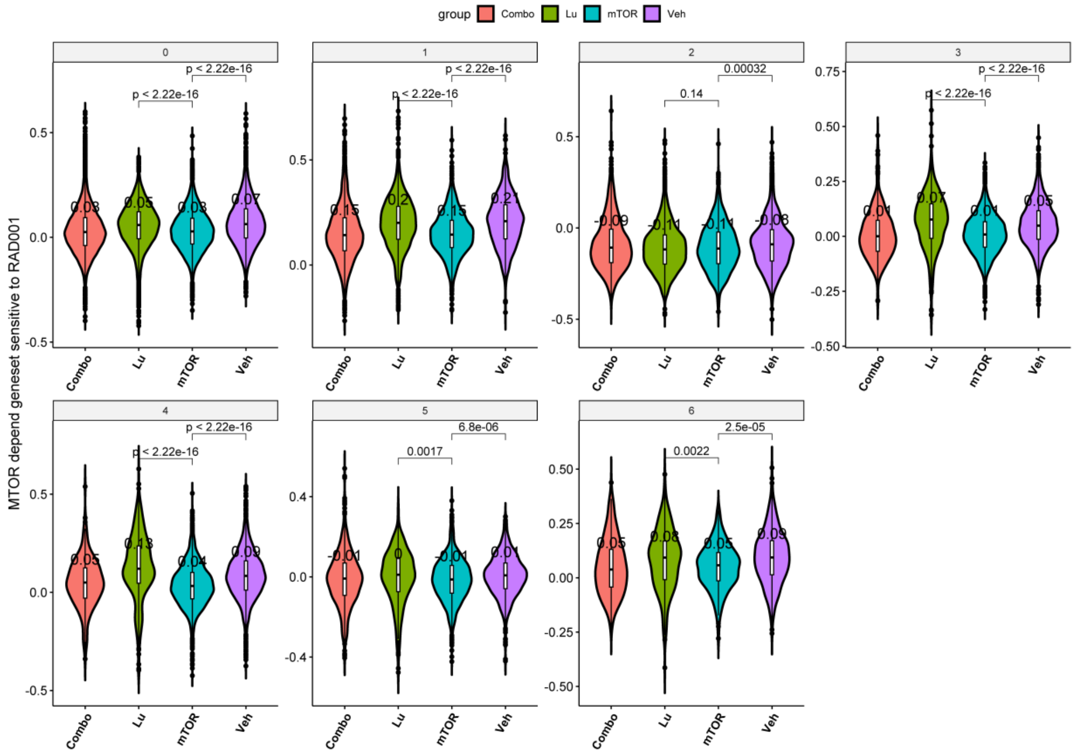

Supplementary Figure S11. Single-cell characterization of RAD001-sensitive mTOR-dependent gene signatures in tumor subclusters. Violin plots derived from single-cell RNA sequencing data illustrate expression levels of the RAD001-sensitive mTOR-dependent gene signature across tumor subclusters; analysis demonstrates that mTOR activity does not directly modulate tumor heterogeneity-driven subclustering, but uniformly suppresses downstream mTOR signaling pathways in all cellular subpopulations.

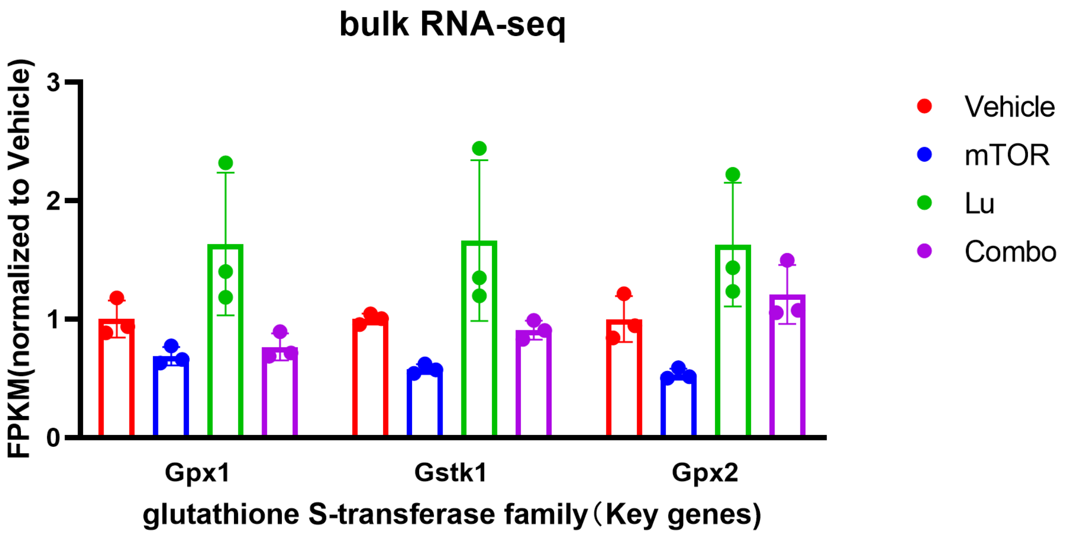

Supplementary Figure S12. Bar plot derived from bulk RNA-seq data demonstrates differential expression patterns of glutathione S-transferase (GST) family members (GSTK1, GPX1, GPX2) across therapeutic interventions (Lu, mTOR, Combo) relative to vehicle control. FPKM values were normalized to the Veh group. These three enzymes were identified as pivotal regulatory nodes in glutathione metabolism.

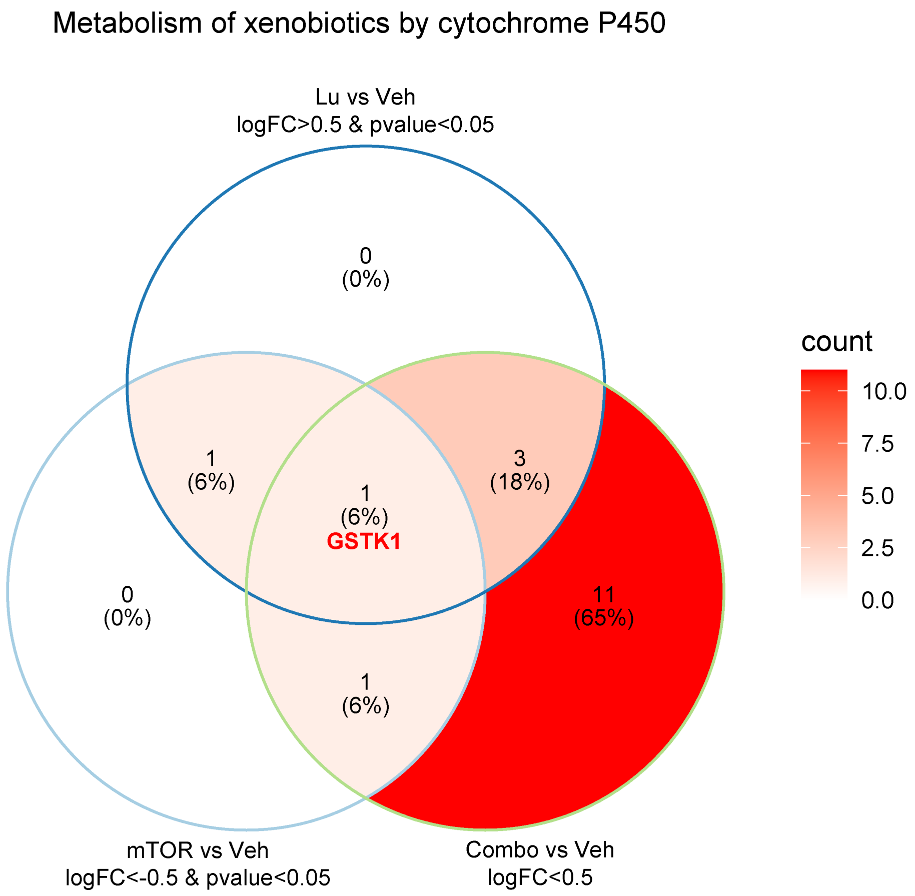

Supplementary Figure S13. Venn diagram depicting overlapping differentially expressed genes (DEGs) in the cytochrome P450 (CYP)-mediated xenobiotic detoxification pathway across Lu vs. Veh, mTOR vs. Veh, and Combo vs. Veh comparisons. GSTK1 was identified as a key regulatory gene within this pathway.

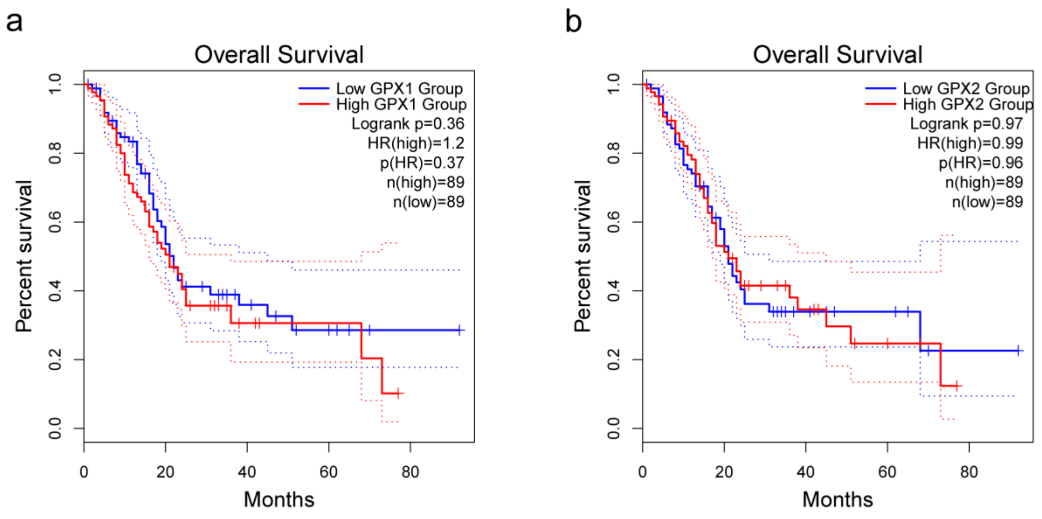

Supplementary Figure S14. Kaplan-Meier survival analysis of (a) GPX1 and (b) GPX2 in pancreatic cancer patients from the TCGA PAAD cohort (n=89). High expression levels of GPX1 (HR=1.2, log-rank p=0.36) and GPX2 (HR=0.99, log-rank p=0.97) showed no statistically significant association with overall survival.

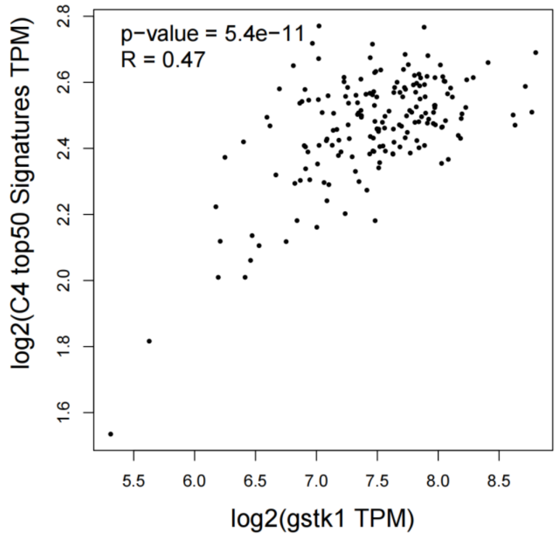

Supplementary Figure S15. Scatter plot demonstrating a moderate-to-strong positive correlation (Pearson’s R = 0.47, $p = 5.4 \times 10^{-11}$) between GSTK1 expression and the aggregated expression of the top 50 genes characterizing the C4 cell population, suggesting GSTK1's potential role in promoting the high-proliferation malignant phenotype of this subpopulation.

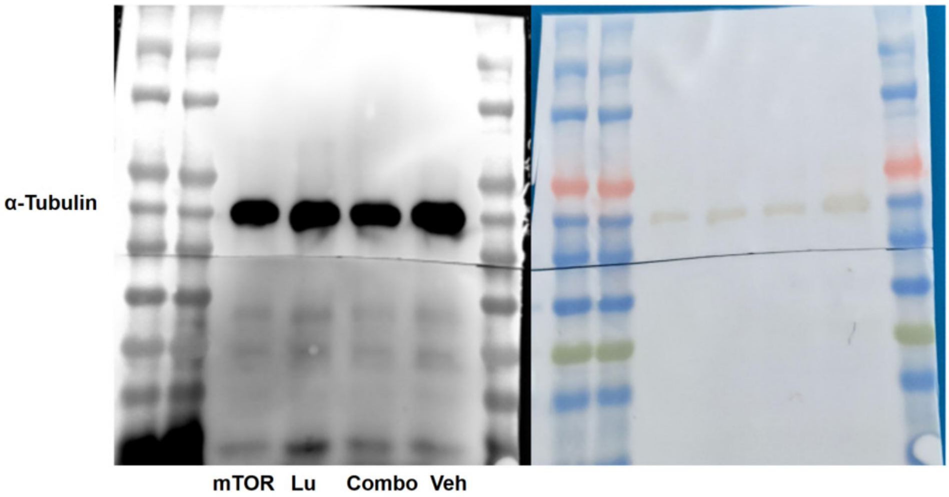

Supplementary Figure S16. Uncropped western blot images showing GSTK1 protein expression in AR42J cells under four treatment conditions: Vehicle control (Veh), [^{177}Lu]Lu-DOTA-CCK$_2$R-dimer (Lu), mTOR inhibitor (mTOR), and combination therapy (Combo).

Supplementary Tables

Supplemental Table 1. Radiolabeling conditions, molar activities, and characterization of CCK2R-targeted tracers.

Supplementary Table 2. Time-dependent *ex vivo* biodistribution of [^{68}Ga]Ga-DOTA-CCK$_2$R-dimer and [^{68}Ga]Ga-DOTA-CCK-66 in AR42J tumor-bearing mice at 0.5, 1, and 2 h post-injection. Data are expressed as mean ± SD (n = 3).

Supplemental Table 3. Tumor-to-background (T/B) ratios of [^{68}Ga]Ga-DOTA-CCK2R-dimer and [^{68}Ga]Ga-DOTA-CCK-66 in AR42J tumor-bearing mice at 0.5, 1, and 2 hours post-injection. Data are presented as mean ± SD (n = 3).

Supplemental Table 4. Time-dependent *ex vivo* biodistribution of [^{177}Lu]Lu-DOTA-CCK$_2$R-dimer in AR42J tumor-bearing mice at 1, 4, 24, 48, 72, and 96 h post-injection. Data are expressed as mean ± SD (n = 3).

Supplemental Table 5. Tumor-to-background (T/B) ratios of [^{177}Lu]Lu-DOTA-CCK$_2$R-dimer in AR42J tumor-bearing mice at 1, 4, 24, 48, 72, and 96 hours post-injection . Data are presented as mean ± SD (n = 3).

Supplemental Table 6. Post-Treatment Biosafety Assessment: Complete Blood Count (CBC) Analysis.

Supplemental Table 7. Post-Treatment Biosafety Assessment: Blood Biochemistry Analysis.

Supplemental Table 1. Radiolabeling conditions, molar activities, and characterization of CCK_2R-targeted tracers

Compound	MW (Da)	Radionuclide	Precursor Amount (µg)	Precursor (nmol)	Activity Used (MBq)	Molar Activity (MBq/nmol)	HPLC Retention Time (min)	Radiochemical Purity (%)
DOTA-CCK-66	1404.7	^{68}Ga	30	21	370	~17.6	12.96	>98
DOTA-CCK_2R-dimer	2534.2	^{68}Ga	30	12	370	~30.8	14.03	>98
DOTA-CCK_2R-dimer	2534.2	^{177}Lu	30	12	370	~30.8	14.02	>98

Supplemental Table 2. Time-dependent *ex vivo* biodistribution of [^{68}Ga]Ga-DOTA-CCK$_2$R-dimer and [^{68}Ga]Ga-DOTA-CCK-66 in AR42J tumor-bearing mice at 0.5, 1, and 2 h post-injection. Data are expressed as mean ± SD (n = 3).

**Organ %ID/g**	**[^{68}Ga]Ga-DOTA-CCK$_2$R-dimer**				**[^{68}Ga]Ga-DOTA-CCK-66**			
**Time**	**0.5 h**	**1 h**	**2 h**	**1 h block**	**0.5 h**	**1 h**	**2 h**	**1 h block**
Heart	1.26 ± 0.44	1.56 ± 0.09	1.14 ± 0.39	0.2 ± 0.15	0.28 ± 0.04	0.89 ± 0.52	0.48 ± 0.31	0.09 ± 0.05
Liver	3.45 ± 1.37	4.24 ± 0.47	2.01 ± 1.19	3.88 ± 2.47	0.74 ± 0.10	1.57 ± 0.22	0.54 ± 0.17	0.96 ± 0.26
Spleen	0.96 ± 0.40	1.03 ± 0.25	0.52 ± 0.05	0.34 ± 0.11	0.71 ± 0.20	1.03 ± 0.60	0.45 ± 0.23	0.34 ± 0.11
Lung	2.01 ± 0.71	2.58 ± 0.85	1.02 ± 0.01	1.28 ± 0.78	0.95 ± 0.14	1.25 ± 0.60	0.87 ± 0.31	0.87 ± 0.89
Kidney	5.24 ± 3.64	11.18 ± 1.84	12.73 ± 3.23	12.32 ± 8.97	1.68 ± 0.19	7.52 ± 1.95	6.4 ± 2.54	12.68 ± 3.71
Stomach	1.11 ± 0.59	2.16 ± 0.10	1.971 ± 0.39	0.54 ± 0.33	0.74 ± 0.27	1.49 ± 0.64	1.63 ± 0.27	0.37 ± 0.23
Small intestine	1.48 ± 0.73	1.64 ± 0.30	0.86 ± 0.32	0.56 ± 0.36	0.57 ± 0.21	0.97 ± 0.39	0.50 ± 0.27	0.27 ± 0.07
Large intestine	1.38 ± 1.37	1.22 ± 0.05	0.67 ± 0.34	0.08 ± 0.04	0.55 ± 0.19	0.65 ± 0.40	0.37 ± 0.06	0.04 ± 0.02
Bladder	0.97 ± 0.52	1.59 ± 0.58	0.53 ± 0.15	0.99 ± 0.77	1.9 ± 1.13	1.59 ± 0.47	1.37 ± 0.43	1.15 ± 1.21
Brain	0.17 ± 0.07	0.21 ± 0.01	0.06 ± 0.04	0.02 ± 0.01	0.03 ± 0.01	0.17 ± 0.04	0.07 ± 0.01	0.02 ± 0.01
Bone	0.78 ± 0.48	0.71 ± 0.17	0.22 ± 0.18	0.03 ± 0.01	0.12 ± 0.05	0.27 ± 0.16	0.1 ± 0.04	0.01 ± 0.003
Muscle	0.57 ± 0.24	0.79 ± 0.15	0.62 ± 0.19	0.15 ± 0.13	0.52 ± 0.27	0.68 ± 0.21	0.72 ± 0.36	0.05 ± 0.04
Tumor	5.81 ± 1.83	12.80 ± 2.05	20.19 ± 3.31	3.91 ± 1.23	3.00 ± 0.63	6.79 ± 1.50	9.53 ± 2.38	2.35 ± 1.18
Blood	2.25 ± 1.20	2.41 ± 1.22	1.56 ± 0.97	0.41 ± 0.26	1.22 ± 0.15	1.08 ± 0.22	0.69 ± 0.30	0.05 ± 0.02

Supplemental Table 3. Tumor-to-background (T/B) ratios of [^{68}Ga]Ga-DOTA-CCK$_2$R-dimer and [^{68}Ga]Ga-DOTA-CCK-66 in AR42J tumor-bearing mice at 0.5, 1, and 2 hours post-injection. Data are presented as mean ± SD (n = 3).

**Organ T/B**	**[^{68}Ga]Ga-DOTA-CCK$_2$R-dimer**			**[^{68}Ga]Ga-DOTA-CCK-66**		
**Time**	**0.5 h**	**1 h**	**2 h**	**0.5 h**	**1 h**	**2 h**
Heart	5.23 ± 2.91	8.18 ± 0.91	18.51 ± 3.74	10.86 ± 1.72	8.93 ± 4.20	33.21 ± 30.19
Liver	2.07 ± 1.51	3.05 ± 0.56	11.88 ± 4.60	4.12 ± 0.63	4.30 ± 0.38	13.21 ± 7.75
Spleen	7.04 ± 3.92	12.64 ± 2.00	39.36 ± 7.60	4.46 ± 1.21	7.00 ± 2.74	24.94 ± 10.95
Lung	3.34 ± 2.07	5.56 ± 2.82	19.72 ± 2.99	3.22 ± 0.56	7.32 ± 5.81	11.71 ± 2.81
Kidney	1.66 ± 1.37	1.15 ± 0.15	1.62 ± 0.29	1.80 ± 0.20	1.10 ± 0.47	1.67 ± 0.79
Stomach	4.50 ± 3.70	5.93 ± 0.89	10.53 ± 2.83	4.50 ± 1.83	5.06 ± 2.32	6.00 ± 1.93
Small intestine	7.72 ± 2.49	8.04 ± 2.02	25.98 ± 11.95	5.83 ± 2.57	7.43 ± 1.49	25.69 ± 17.86
Large intestine	8.98 ± 3.85	10.5 ± 1.55	40.21 ± 29.42	5.91 ± 1.81	13.26 ± 6.96	26.22 ± 5.50
Bladder	7.42 ± 4.09	8.41 ± 1.60	40.78 ± 16.87	1.95 ± 0.96	4.41 ± 1.16	7.50 ± 2.91
Brain	39.67 ± 24.61	61.79 ± 7.46	153.87 ± 17.46	72.83 ± 22.60	91.41 ± 5.94	140.63 ± 66.32
Bone	19.23 ± 4.91	18.81 ± 5.04	141.91 ± 25.04	29.95 ± 14.14	34.88 ± 29.42	98.04 ± 19.94
Muscle	11.78 ± 5.75	16.54 ± 2.99	33.03 ± 7.91	6.69 ± 2.78	10.25 ± 1.41	15.20 ± 6.94
Blood	3.01 ± 1.29	6.80 ± 4.41	21.58 ± 9.41	2.48 ± 0.27	6.36 ± 1.30	18.12 ± 14.89

Supplemental Table 4. **Time-dependent *ex vivo* biodistribution of [^{177}Lu]Lu-DOTA-CCK$_2$R-dimer in AR42J tumor-bearing mice at 1, 4, 24, 48, 72, and 96 hours post-injection. Data are expressed as mean±SD (n=3).**

**Organ %ID/g Time**	**1 h**	**4 h**	**24 h**	**48 h**	**72 h**	**96 h**
Heart	1.29 ± 0.41	0.67 ± 0.34	0.05 ± 0.03	0.02 ± 0.02	0.02 ± 0.008	0.03 ± 0.01
Liver	4.44 ± 1.80	3.42 ± 0.75	0.61 ± 0.11	0.58 ± 0.16	0.37 ± 0.05	0.42 ± 0.11
Spleen	2.62 ± 1.02	2.23 ± 0.32	0.15 ± 0.08	0.02 ± 0.01	0.05 ± 0.03	0.15 ± 0.09
Lung	2.40 ± 0.05	1.06 ± 0.39	0.04 ± 0.03	0.02 ± 0.01	0.01 ± 0.01	0.02 ± 0.005
Kidney	8.92 ± 1.05	6.79 ± 1.58	3.43 ± 0.84	1.59 ± 1.00	1.60 ± 0.39	1.00 ± 0.50
Stomach	3.88 ± 0.66	3.05 ± 0.60	1.07 ± 0.28	1.04 ± 0.17	0.26 ± 0.11	0.24 ± 0.30
Small intestine	1.45 ± 0.39	0.58 ± 0.31	0.06 ± 0.04	0.06 ± 0.01	0.02 ± 0.01	0.04 ± 0.03
Large intestine	1.14 ± 0.38	0.61 ± 0.03	0.07 ± 0.01	0.05 ± 0.02	0.04 ± 0.03	0.05 ± 0.03
Bladder	0.84 ± 0.31	0.40 ± 0.13	0.18 ± 0.04	0.03 ± 0.01	0.06 ± 0.03	0.10 ± 0.05
Brain	0.14 ± 0.01	0.03 ± 0.01	0.003 ± 0.002	0.003 ± 0.002	0.004 ± 0.003	0.006 ± 0.002
Bone	0.65 ± 0.27	0.35 ± 0.19	0.04 ± 0.02	0.004 ± 0.001	0.03 ± 0.02	0.02 ± 0.01
Muscle	0.36 ± 0.10	0.16 ± 0.17	0.01 ± 0.01	0.007 ± 0.003	0.007 ± 0.002	0.006 ± 0.004
Tumor	19.17 ± 8.43	14.73 ± 2.83	6.12 ± 1.45	4.32 ± 0.75	2.01 ± 0.47	1.10 ± 0.04
Blood	6.93 ± 1.84	1.19 ± 0.30	0.05 ± 0.01	0.02 ± 0.01	0.002 ± 0.0005	0.008 ± 0.007

Supplemental Table 5. Tumor-to-background (T/B) ratios of [^{177}Lu]Lu-DOTA-CCK$_2$R-dimer in AR42J tumor-bearing mice at 1, 4, 24, 48, 72, and 96 hours post-injection . Data are presented as mean ± SD (n = 3).

Organ<br>T/B<br>Time	1 h	4 h	24 h	48 h	72 h	96 h
Heart	15.31 ± 5.59	29.59 ± 12.81	133.89 ± 59.91	405.51 ± 29.22	80.50 ± 26.44	105.07 ± 5.19
Liver	5.19 ± 2.80	4.37 ± 0.90	10.00 ± 0.99	7.78 ± 1.96	5.53 ± 1.05	2.70 ± 0.63
Spleen	8.21 ± 4.18	6.59 ± 0.75	55.00 ± 21.08	123.86 ± 50.01	51.84 ± 19.48	9.63 ± 3.59
Lung	7.93 ± 3.29	15.91 ± 6.64	331.41 ± 35.03	900.02 ± 88.01	298.53 ± 66.52	61.17 ± 12.81
Kidney	2.22 ± 1.01	2.19 ± 0.23	1.84 ± 0.56	4.16 ± 3.43	1.65 ± 0.05	1.37 ± 0.84
Stomach	4.83 ± 1.46	5.05 ± 1.72	5.94 ± 1.92	4.15 ± 0.31	8.41 ± 1.18	13.36 ± 6.42
Small intestine	14.01 ± 5.39	28.78 ± 10.31	228.80 ± 10.04	111.63 ± 61.04	186.59 ± 27.73	39.57 ± 12.19
Large intestine	17.14 ± 5.58	24.43 ± 5.51	90.67 ± 23.41	73.24 ± 14.51	98.87 ± 74.36	26.32 ± 11.03
Bladder	24.75 ± 11.01	40.41 ± 12.13	35.43 ± 6.48	193.53 ± 91.05	44.73 ± 13.13	13.50 ± 4.12
Brain	141.47 ± 50.01	470.08 ± 81.07	6080.06 ± 100.02	1706.81 ± 150.63	746.21 ± 85.13	188.54 ± 50.12
Bone	35.13 ± 13.61	53.25 ± 32.63	164.65 ± 44.96	1069.10 ± 247.06	116.81 ± 40.12	45.96 ± 9.49
Muscle	53.32 ± 18.19	246.45 ± 180.08	417.18 ± 209.62	531.15 ± 153.36	347.84 ± 238.07	90.85 ± 32.76
Blood	2.95 ± 1.50	13.27 ± 5.68	140.98 ± 51.28	185.72 ± 14.28	767.72 ± 140.13	225.66 ± 40.93

Supplemental Table 6. Post-Treatment Biosafety Assessment: Complete Blood Count (CBC) Analysis. Data are expressed as mean ± SD (n = 3).

Items	Control	^{177}Lu	RAD001	Combination
WBC (10^9/L)	7.67 ± 2.52	7.0 ± 2.65	7.67 ± 1.15	7.33 ± 1.53
LYM# (10^9/L)	0.39 ± 0.16	0.38 ± 0.14	0.41 ± 0.03	0.37 ± 0.15
MON# (10^9/L)	0.16 ± 0.04	0.15 ± 0.13	0.17 ± 0.05	0.20 ± 0.09
GRA# (10^9/L)	0.22 ± 0.06	0.20 ± 0.07	0.19 ± 0.05	0.22 ± 0.06
LYM (%)	49.37 ± 6.24	52.27 ± 2.74	53.9 ± 5.47	49.1 ± 5.62
MON (%)	22.27 ± 3.92	19.03 ± 9.18	22.13 ± 2.32	25.27 ± 3.64
GRA (%)	28.37 ± 2.35	28.7 ± 8.16	23.97 ± 4.05	26.63 ± 5.59
HGB (g/L)	183.03 ± 4.78	151.67 ± 19.64	178.57 ± 3.40	176.73 ± 9.23
MCH (pg)	33.87 ± 1.02	41.83 ± 6.37	35.33 ± 0.87	36.37 ± 1.59
MCHC (g/L)	669.67 ± 19.04	714 ± 42.04	693.67 ± 15.95	711 ± 47.70
RBC (10^{12}/L)	5.4 ± 0.30	4.72 ± 0.81	5.05 ± 0.22	4.9 ± 0.69
MCV (fL)	50.67 ± 0.47	51.63 ± 0.81	51.03 ± 0.57	51.8 ± 0.2
HCT (%)	27.33 ± 1.36	19.2 ± 5.20	25.73 ± 1.05	25.57 ± 2.97
RDWSD (fL)	22.33 ± 1.15	22.33 ± 1.15	23.33 ± 1.10	23.33 ± 1.20
RDWCV (%)	10.97 ± 0.59	10.77 ± 0.40	11.23 ± 0.15	11.13 ± 0.12
PLT (10^9/L)	111.33 ± 7.51	116 ± 13.08	104.33 ± 2.52	114 ± 9.54
PCT (%)	0.12 ± 0.04	0.16 ± 0.04	0.12 ± 0.01	0.14 ± 0.05
MPV (fL)	13.3 ± 0.70	12.2 ± 0.61	13.8 ± 0.35	13.63 ± 0.25
PDW (fL)	32.07 ± 1.19	28.67 ± 2.04	32.9 ± 0.3	30.73 ± 2.89
P-LCR (%)	42.73 ± 4.53	35.87 ± 5.49	45.87 ± 1.03	45 ± 3.03

The table provides the abbreviations and their corresponding full names for commonly used blood test parameters: White Blood Cell (WBC), Lymphocyte (LYM#), Monocyte (MON#), Granulocyte (GRA#), Lymphocyte Percentage (LYM%), Monocyte Percentage (MON%), Granulocyte Percentage (GRA%), Hemoglobin (HGB), Mean Corpuscular Hemoglobin (MCH), Mean Corpuscular Hemoglobin Concentration (MCHC), Red Blood Cell (RBC), Mean Corpuscular Volume (MCV), Hematocrit (HCT), Red Cell Distribution Width (RDWSD, RDWCV), Platelet (PLT), Plateletcrit (PCT), Mean Platelet Volume (MPV), Platelet Distribution Width (PDW), and Platelet Large

Cell Ratio (P-LCR).

Supplemental Table 7. Post-Treatment Biosafety Assessment: Blood Biochemistry Analysis.Data are expressed as mean ± SD (n = 3).

**Items**	**Control**	**^{177}Lu**	**RAD001**	**Combination**
ALP (U/L)	167.68 ± 25.59	135.99 ± 24.61	150.68 ± 32.99	145.59 ± 2.00
ALT (U/L)	43.71 ± 6.25	44.93 ± 6.41	43.73 ± 11.08	39.23 ± 2.69
AST (U/L)	109.69 ± 14.37	87.94 ± 5.64	109.76 ± 13.94	101.56 ± 23.11
UA (µmol/L)	132.61 ± 49.17	126.33 ± 13.85	140.92 ± 41.88	124.69 ± 9.76
UREA (mmol/L)	4.10 ± 0.72	3.25 ± 0.56	3.49 ± 1.30	3.9 ± 0.68

The table lists the abbreviations and their corresponding full names for common blood biochemical markers: Alanine Aminotransferase (ALT), Aspartate Aminotransferase (AST), Alkaline Phosphatase (ALP), Uric Acid (UA), and Urea (UREA).
